# Predicting translational progress in biomedical research

**DOI:** 10.1371/journal.pbio.3000416

**Published:** 2019-10-10

**Authors:** B. Ian Hutchins, Matthew T. Davis, Rebecca A. Meseroll, George M. Santangelo

**Affiliations:** Office of Portfolio Analysis, Division of Program Coordination, Planning, and Strategic Initiatives, National Institutes of Health, Bethesda, Maryland, United States of America; McGill University, CANADA

## Abstract

Fundamental scientific advances can take decades to translate into improvements in human health. Shortening this interval would increase the rate at which scientific discoveries lead to successful treatment of human disease. One way to accomplish this would be to identify which advances in knowledge are most likely to translate into clinical research. Toward that end, we built a machine learning system that detects whether a paper is likely to be cited by a future clinical trial or guideline. Despite the noisiness of citation dynamics, as little as 2 years of postpublication data yield accurate predictions about a paper’s eventual citation by a clinical article (accuracy = 84%, F1 score = 0.56; compared to 19% accuracy by chance). We found that distinct knowledge flow trajectories are linked to papers that either succeed or fail to influence clinical research. Translational progress in biomedicine can therefore be assessed and predicted in real time based on information conveyed by the scientific community’s early reaction to a paper.

## Abstract 2

Fundamental scientific advances can take decades to translate into improvements in human health. This study shows that a machine learning model can accurately predict whether an article is likely to be cited by a future clinical trial or guideline, using as little as two years of post-publication citation data.

## Introduction

Much of the recent discussion of bench-to-bedside research has appropriately been focused on how best to allocate limited resources in support of the science that gives rise to transformative clinical impact. Making these decisions is complicated by the fact that it can take decades for a fundamental discovery to translate into improvements in human health [1–11]. Current initiatives are targeting key barriers, both scientific and operational, that inhibit or delay bench-to-bedside translation [12]. To date, however, these efforts have not attempted to address translational barriers by improving our understanding of the mechanism(s) through which new scientific knowledge is transmitted from research outputs into the clinic. Characterizing these pathways of knowledge flow, defined as the movement of information from cited articles to citing articles, might have the additional benefit of identifying otherwise unnoticed discoveries that are good candidates for bench-to-bedside translation.

In the past, tracking knowledge flow from bench to bedside has required manual curation by subject matter experts [1–3,13,14]. Although informative, those types of analyses are time intensive and thus cannot be used to analyze large data sets. This limitation is disappearing due to recent advances in algorithm development and data availability, which now present the opportunity to measure the progress of biomedical research at scale [15]. Early efforts along these lines have relied upon a single variable curated by experts to position large groups of papers along the fundamental/clinical research axis [4,16]. The extension of this approach to high-throughput, multidimensional analyses has the potential to identify commonalities shared by past discoveries that had clinical impact, which in turn could be leveraged to determine the likelihood that a recent discovery will have future translational success. This information could assist scientists and administrators as they attempt to address obstacles to translation [3].

A priori, any attempt to determine the translational potential of a recent discovery faces 2 key challenges. First, although it is a subject of some debate, individual scientific advances are generally considered to be unpredictable. Vannevar Bush, in *Science*, *the Endless Frontier* [5], said, “Statistically it is certain that important and highly useful discoveries will result from some fraction of the undertakings in basic science; but the results of any one particular investigation cannot be predicted with accuracy.” Second, scholarly citations accrue in a noisy fashion, thereby complicating efforts to trace the flow of knowledge. More optimistically, recent work has shown that citation dynamics, and therefore the underlying knowledge flow among scientists, obey fundamental mathematical principles [17]. This raises the question: can modern predictive analytics be used to identify the subset of “particular investigations” that have a high potential for translation?

We report here the development of a method to predict translational progress computationally at the article level. We trained machine learning models on a binary output—whether or not a clinical trial or guideline (hereafter referred to collectively as clinical articles) eventually cited the article of interest, recognizing that most of these translational “shots on goal” do not directly “score” an improvement in human health. Despite the noisiness of citation dynamics, 2 years of postpublication data suffice for the model to predict accurately whether a research paper will eventually be cited by a clinical article. As information continues to accumulate in growing citing networks [18], predictive power increases until it reaches a plateau approximately 7 years post publication, indicating that knowledge transfer is most active during this initial 7-year period. Prediction confidence correlates with eventual citation by a clinical article, suggesting that this measure serves as an early signature of translation. Furthermore, we have identified a subset of citation patterns, which can be thought of as knowledge flow trajectories, that occur frequently and presage either successful or delayed translation. Taken together, these findings demonstrate that the scientific community’s early reaction to a paper provides sufficient information for a machine learning system to predict translational progress in biomedicine.

## Results

### Temporal dynamics of translational science

Before attempting to predict translation, we first needed to define it. Citations by published clinical articles are the most commonly used measure of translational progress; however, the relationship between early scientific discoveries and the cures to which they eventually lead is complex [14]. One facet of this complexity is that important early steps in translation may appear topically distinct from their clinical descendants, raising the possibility that this method of evaluating the movement of ideas from bench to bedside might undervalue the subset of transformative biomedical research that lacks a direct link to human health. To test this possibility, we used information on the Nobel website (www.nobelprize.org) to identify seminal papers that earned the Prizes in Physiology or Medicine between 1995 and 2018; all but the discovery of circadian rhythm (which was awarded the Prize in 2017; see Discussion) were first reported in publications that have since been cited by at least one and as many as 159 clinical articles (Table 1). Using citations by clinical articles as evidence of translational progress therefore does not disadvantage transformative discoveries that have laid the foundation for later clinical work; when they impact human health, papers describing those discoveries are recognized and cited directly by clinical researchers.

**Table 1:** Seminal publications leading to Nobel Prizes in Physiology or Medicine and their clinical citations.

Topic	Prize year	Publication date	PMID^1^	Total citations	RCR^2^	RCR percentile^3^	Citations by clinical articles
Immunotherapy	2018	1996	8596936	1,126	19.2	99.7	48
Circadian rhythms	2017	April 1984	16593450	82	2.1	76.3	0
		October 1984	6435882	149	3.8	89.9	0
		December 1984	6094014	96	2.2	77.6	0
		December 1984	6440029	135	3.3	87.4	0
Autophagy	2016	1992	1400575	523	13.5	99.0	1
Treatment of parasitic diseases	2015	1979	464563	132	6.6	96.1	7
Positioning system in the brain	2014	1971	5124915	1,773	60.4	99.9	14
Vesicle trafficking	2013	1993	8455717	1,776	51.6	99.9	1
Reprogramming mature cells to pluripotency	2012	2007	18035408	7,890	238.8	99.9	17
Activation of the immune system	2011	1998	9851930	3,976	113.4	99.9	21
In vitro fertilization	2010	1978	79723	680	34.4	99.8	33
Telomeres and telomerase	2009	1978	642006	407	10.9	98.5	2
HPV/cervical cancer and HIV/AIDS	2008	1983	6304740	913	31.5	99.8	8
		1983	6189183	2,849	102.4	99.9	32
Introducing specific gene modifications in mice	2007	1987	2822260	1,180	30.0	99.8	1
RNA interference	2006	1998	9486653	6,500	163.5	99.9	7
*Helicobacter pylori* and ulcers	2005	1984	6145023	2,285	95.8	99.9	100
Olfactory biology	2004	1991	1840504	1,953	60.2	99.9	14
MRI	2003	1984	6571566	232	14.0	99.1	7
Genetic regulation of organ development and apoptosis	2002	1977	838129	1,511	33.0	99.8	1
Regulators of the cell cycle	2001	1987	3553962	576	14.7	99.2	2
Signal transduction in the nervous system	2000	1958	13529006	237	9.6	98.1	4
Protein transport	1999	1975	811671	1,678	57.9	99.9	1
Nitric oxide signals in the cardiovascular system	1998	1980	6253831	5,824	330.2	99.9	159
Prions	1997	1982	6801762	2,349	77.5	99.9	5
Cell-mediated immune defense	1996	1974	4133807	1,003	29.6	99.8	4
Genetic control of early embryonic development	1995	1980	6776413	1,701	42.0	99.9	2

^**1**^Identification number assigned to each article indexed in PubMed.

^2^A field- and time-normalized measure of article influence [18].

^3^Percentile rank based on RCR values of NIH-funded articles.

**Abbreviations:** HPV, human papilloma virus; PMID, PubMed Identifier; RCR, Relative Citation Ratio

**Fig 1:**
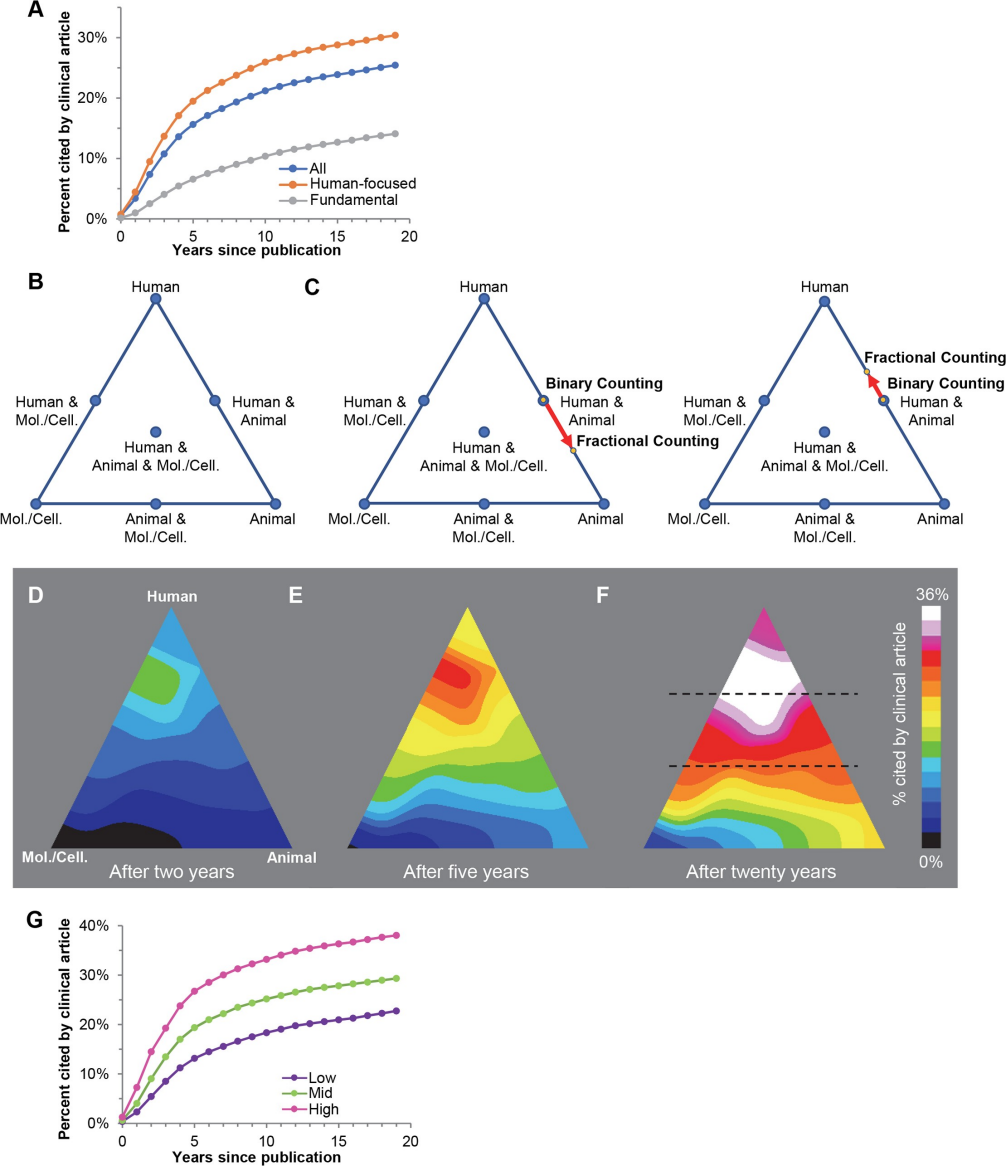
Temporal dynamics of translation. (A) Cumulative proportion of publications cited by a clinical article over time for all articles (blue), human-focused articles (orange), and fundamental articles (grey). (B) Trilinear graph illustrating the triangle of biomedicine described by Weber [4]. Articles in PubMed are classified as Human, Animal, or Molecular/Cellular—or as a combination of these—based on their MeSH terms. (C) Locations of 2 publications that are more accurately plotted on the Human–Animal axis of the trilinear graph due to fractional counting. The publication plotted on the left triangle (PMID 25638260) has 1 Human term and 3 Animal terms, while the publication plotted on the right (PMID 27565847) has 5 Human terms and 3 Animal terms. With binary counts, both are located at the center of the Human–Animal axis, but with fractional counting they are shifted toward the Animal and Human vertices, respectively. (D–F) Density graph of the percentage of papers across the trilinear graph cited by clinical articles 2, 5, or 20 years after publication. (G) Cumulative proportion of publications cited by a clinical article over time for articles with intermediate Human fractional counts (greater than 0% and less than 100%): low (purple; ≤33.3% Human; below lower dashed line in panel F), mid (green; >33.3%, ≤66.7%; between dashed lines in panel F), and high (pink; >66.7%; above upper dashed line in panel F). MeSH, Medical Subject Headings; Mol./Cell., Molecular/Cellular; PMID, PubMed Identifier.

Citations by clinical articles have the additional advantage of being readily quantifiable [1,19]. Since each of the approximately 225,000 clinical articles published between 1995 and 2005 cited an average of 25 references (a total of approximately 5.6 million citations), theoretically nearly all approximately 6 million PubMed articles published during that same timeframe could have been cited by a clinical article. Tracking all PubMed research articles in a single year (1995), and following their accumulation of citations by clinical articles over a 20-year period (end of 1995–2014), shows that, in reality, only 25% of papers meet this mark (Fig 1A; blue line). Whether a publication will receive a clinical citation varies by its article type: reviews are most likely to receive a clinical citation (26.1%), followed by research articles (18.3%) and nonresearch nonreview articles (e.g., front matter, 6.7%). The number of all articles cited by a clinical trial is more accurately captured by removing the roughly one-fifth of papers that received no citations of any kind from the denominator; doing so increases the percentage of articles for which there is evidence of translational success to 30%.

The prediction of future clinical citations based on past citation history can only be successful if that history follows detectable patterns. While this is a reasonable hypothesis, to our knowledge it has not previously been tested. To determine whether the 30% of publications that received clinical citations share any common features, we first asked whether the likelihood a paper will be cited by a clinical article can be predicted by the overall number of citations the paper receives, recognizing that this approach has the disadvantage of being slow because citations take time to accumulate. In the aggregate, counting total citations does have at least some predictive power, because publications cited by a clinical article are more highly cited overall than their counterparts (a median of 28 versus 8 total citations, respectively; S1A Fig). However, for any individual paper, total citations are not a strong predictor of eventual clinical citation. For example, the chance of being cited by a clinical article is roughly 50/50 for papers receiving between 30 citations and 100 citations. More importantly, 14.9% of papers with 8 total citations are cited by a clinical article. The number of citations a paper receives is therefore by itself an insufficiently accurate predictor of future citation by a clinical article.

Analyzing the content of the papers cited only 8 times that nevertheless exhibited translational progress revealed topics predominantly focused on human diseases and therapies (S1B Fig). This suggested the possibility that measuring 2 parameters, total citations and the degree to which the research is human focused, might be sufficient in predicting future citation by a clinical article. Alternatively, a more sophisticated approach might be required. To begin distinguishing between these ideas, we compared the rates at which clinical citations accrue for 2 distinct sets of papers—those that describe either human-focused or fundamental research (Fig 1A, orange and grey lines, respectively). We identified human-focused papers as those for which all relevant terms fell within the human branch of the Medical Subject Headings (MeSH) ontology (assigned by National Library of Medicine [NLM] indexers [20], and see below), as described by Weber [4]. For comparison, we defined fundamental papers as those with no relevant terms that fell within the Human branch of the MeSH tree. Note that referring to papers with no Human MeSH terms as “fundamental” is an operational definition used for convenience and should not be interpreted as a judgment about the underlying nature or goals of the science described therein. Whereas human-focused papers display a rapid increase in the percentage cited by at least one clinical article, fundamental papers ramp up more slowly; within 20 years after publication, human-focused studies are twice as likely as fundamental studies to be cited by a clinical article (30.4% versus 14.1%, respectively). Though this confirmed that measuring total citations and human focus represents an improved way to predict translational progress, it also suggested that the relationship between these two parameters and clinical citations is not simple and that a more sophisticated approach was needed.

To capture the translational potential of publications across the entire biomedical research landscape, we adapted an algorithm that maps papers on a trilinear graph by using 3 overarching branches of the MeSH ontology: Human, Animal, and Molecular/Cellular (HAMC; “the triangle of biomedicine” [4]; Fig 1B). Almost all NIH-funded papers (>96%) are assigned at least one HAMC MeSH term and can be plotted somewhere on the triangle. We modified the algorithm so that the HAMC categories are fractionally counted (Fig 1C), which is done for each article by dividing the number of HAMC terms in each category by the total number of terms in all 3 categories. This results in a major improvement relative to binary counts, both in resolution when plotting on the trilinear graph (Fig 1C) and in the degree of correlation with assessments by human curators (for full details and additional visualizations, see S1 Text and S2 Fig and S1 Movie).

Fig 1D–1F and S2 Movie show the relative density of publications in each region of the triangle of biomedicine that were cited by one or more clinical articles over time. Consistent with the data in Fig 1A, fundamental papers along the base of the triangle (those with 0% Human MeSH terms) require a longer interval for clinical citations to accrue and receive relatively few of those citations overall. Dividing the triangle into thirds (Fig 1F, dashed lines) illustrates the increasing likelihood of translational success for papers that map closer to the Human vertex (Fig 1G). Interestingly, papers in the top third of the triangle (Fig 1G, pink line) plateau at a higher level than those that map at the Human vertex (38.0% versus 30.4%, respectively; Fig 1A). The “hottest” part of the triangle is the region just below the Human vertex, suggesting that papers with a predominantly but not exclusively human focus are most likely to translate. This trend persists even when excluding publications with only one MeSH term, which by definition populate one of the vertices in the triangle of biomedicine (S3 Fig). The fact that there are clearly delineated “hot” and “cold” regions across the trilinear graph suggested that we could exploit this pattern in making predictions about the likelihood that any given paper will be cited by a clinical article. Together, these results point to likely markers of translational progress hidden in the complex combination of content and citation dynamics.

### Using machine learning to predict bench-to-bedside translation

**Fig 2:**
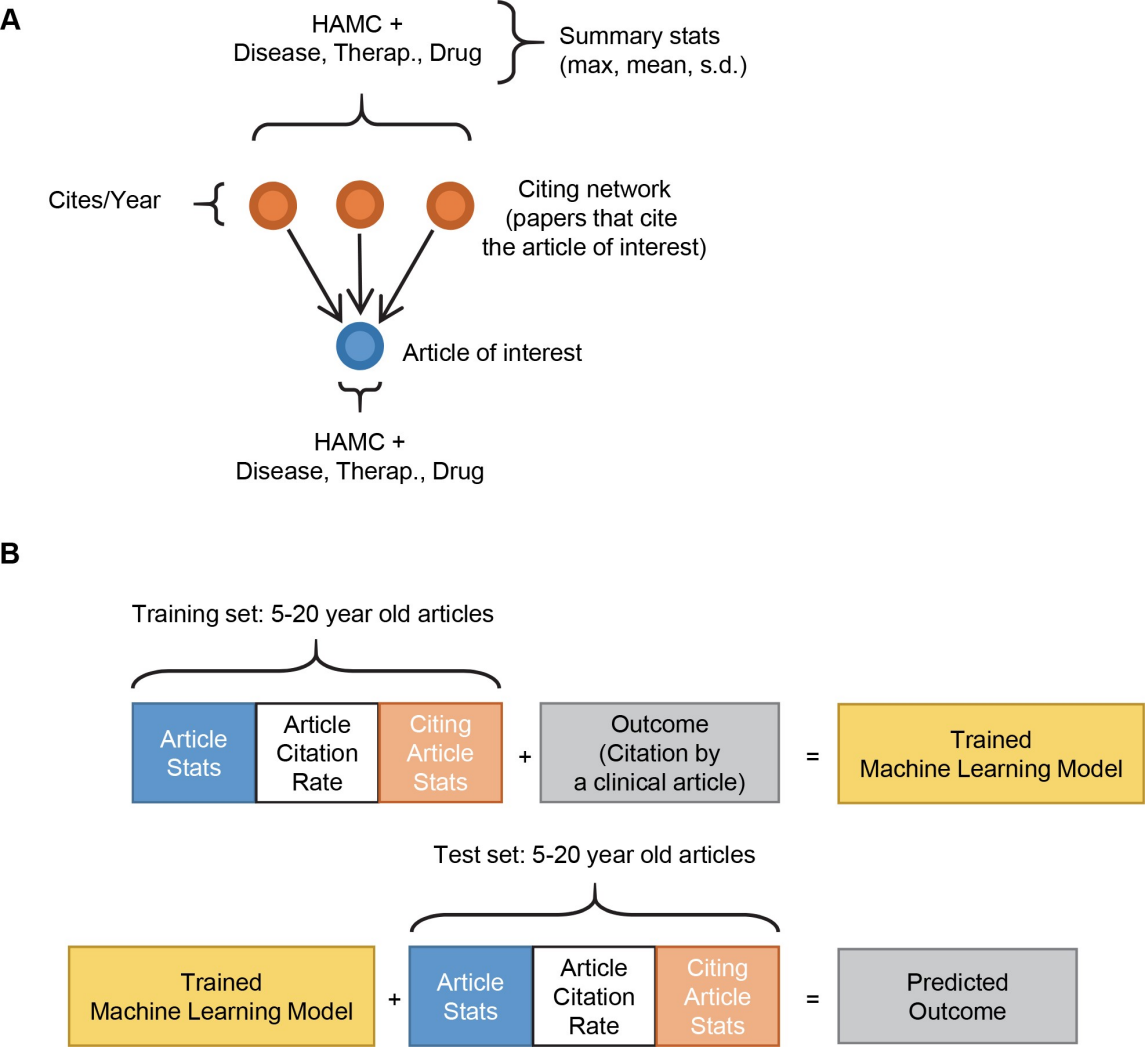
Training a machine learning system to predict future translation. (A) Schematic of inputs into the machine learning system. Papers were assigned the same HAMC scores used for visualization on the trilinear graphs, as well as 3 binary indicators, one each for the presence of modifying MeSH terms in either the Disease, Therapeutic/Diagnostic Approaches, or Chemical/Drug categories. These properties were also scored for papers citing the article of interest, and the citing network was summarized by the max, mean, and standard deviations (SDs), as well as the overall citation rate (cites/year). For this analysis, citation rate is preferable to RCR [18], because citations per year can be used immediately while a meaningful citation count must accrue before RCR can be calculated. (B) Schema for training the machine learning model and generating predictions. HAMC, Human, Animal, and Molecular/Cellular; max, maximum; MeSH, Medical Subject Headings; RCR, Relative Citation Ratio.

The data in Fig 1 suggested the existence of generalizable markers associated with the subset of papers that are eventually cited by one or more clinical articles. We wondered whether such markers might appear early enough in the bench-to-bedside translation process to allow accurate prediction of the clinical utility of a paper within the first few years after its publication. Machine learning is well suited to address that type of question. We developed a model that incorporated the data available for each paper: its citation rate, one feature for each of the 6 the categories of MeSH terms representing its content (HAMC, plus 3 modifying MeSH term categories: Chemical/Drug, Disease, and Therapeutic/Diagnostic Approaches), and 15 summary statistics representing the collection of MeSH terms that map to the papers that cite it (Fig 2A). These 22 features were transformed into vectors for the machine learning system (see Methods for details); we define this multidimensional array of information for each paper in PubMed as its data profile. Vector information for each data profile is supplied to the machine learning system during the training phase alongside an output variable, in this case a binary flag indicating whether or not the paper was cited by a clinical article (Fig 2B, top row). After the training phase, data profiles of a test set (i.e., out-of-sample articles that were excluded from the training set) were evaluated by the system to determine how likely those papers were to receive a clinical citation (Fig 2B, bottom row).

**Fig 3:**
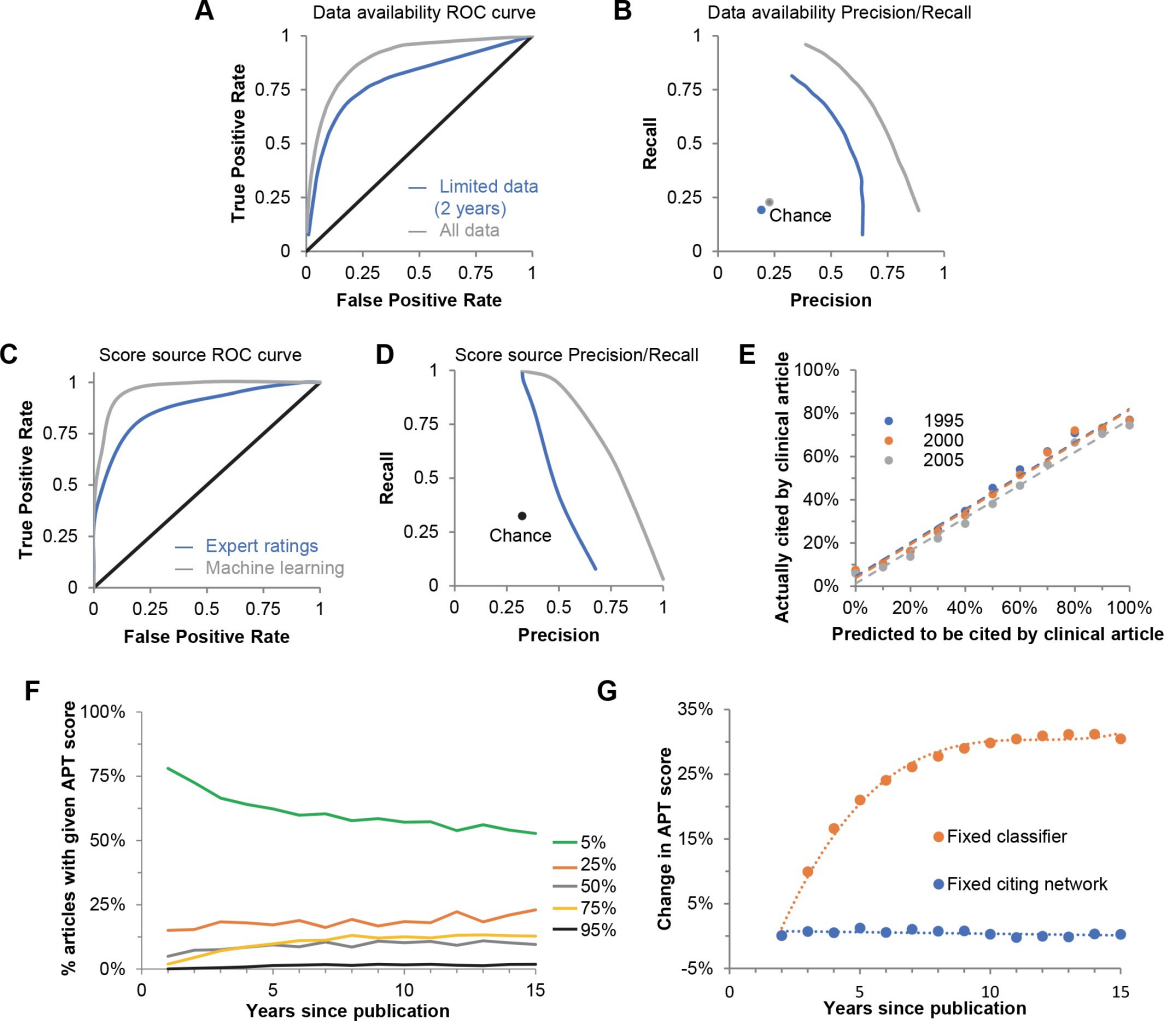
Validation of machine learning predictions. (A, B) Prediction performance for citation data limited to the first 2 years post publication (blue line) or including all data available at the time of the analysis (grey line). (A) Receiver operating characteristic curve to assess performance of predictions (chance, black line). AUC: 0.80 (blue line) and 0.90 (grey line). (B) Precision-recall graphs to assess performance (chance, blue or grey dot). Accuracy: 84% (blue line), 85% (grey line); accuracy is defined as the percent of test set predictions that were correct. F1 scores: 0.56 (blue line), 0.68 (grey line). (C, D) Postpublication peer-review scores from Hutchins and colleagues ([18]; blue line) were compared with machine learning prediction scores (grey line); reviewers rated clinical impact on a scale of 1–5 (*N =* 985 rating scores). (C) Receiver operating characteristic curve to assess performance of expert scores and machine learning predictions (chance, black line). AUC: 0.64 (blue line) and 0.85 (grey line). (D) Precision-recall graphs to assess performance. Accuracy: 69% (blue line), 81% (grey line); F1 scores: 0.52 (blue line), 0.68 (grey line). (E) Correlation between predicted and actual citations for papers published in 1995 (blue), 2000 (orange), and 2005 (grey), using 2 years of citing network data for predictions. Dashed lines represent a linear regression; the slope and r^2^ values, respectively, for each year are as follows: 1995 (0.775, 0.985), 2000 (0.779, 0.984), and 2005 (0.759, 0.988). (F) Percent of articles published in 2000 falling into each of the 5 APT score bins over time. (G) APT scores are assigned based on new information entering the citing network of individual papers. At increasing time intervals post publication, the change in APT score was determined either for classifiers trained on data available in each year (blue) or for a fixed classifier trained on the growing citing networks (orange). APT, Approximate Potential to Translate; AUC, area under the curve; ROC, receiver operating characteristic.

If the machine learning system could make accurate predictions about citation by a clinical article relatively soon after a paper is published, it would indicate that the early stages of knowledge flow are predictive of translational endpoints. We therefore asked what degree of predictive accuracy was possible if data profiles were strictly limited to information available within 2 years after each paper is published. Because papers 5 to 20 years old have a relatively stable percentage of clinical citations (Fig 1A), we selected that timeframe for our training set. Of the different algorithms we tested (Random Forests, Support Vector Machines, logistic regression, Maxent, LibLinear, and Neural Networks; see Methods), Random Forests [21] achieved the highest level of accuracy (84%), a substantial improvement over chance (19%). The corresponding F1 score (harmonic mean of recall and precision; [22]) was 0.56 (Fig 3A and 3B, blue lines). Information limited to what is available within 2 years post publication therefore carries surprisingly strong predictive power about future citations by clinical articles.

We next tested the accuracy of machine learning predictions when papers’ data profiles included their entire citing networks, rather than limiting to citations within the first 2 years after publication (Fig 3A and 3B, grey and blue lines, respectively). This resulted in a slight improvement in accuracy (to 85%; chance = 23%), and the F1 score improved from 0.56 to 0.68. We added further information to our data profiles, such as the historical percentage of clinical citations received by papers in the same journal, the historical percentage of clinical citations to all papers that used the same animal model as the article of interest, and the article’s mention of a Food and Drug Administration (FDA)-approved drug. This resulted in an F1 score of 0.70. Given the additional computational complexity of these features and the modest increase in F1 score, we did not include them in subsequent analyses. We also tested predictions of article features alone, regardless of their citing networks. Including only the article HAMC MeSH terms in the model resulted in an F1 score of 0.08; adding Chemical/Drug, Disease, and Therapeutic/Diagnostic Approaches MeSH terms raised the F1 score to 0.36, still far below the full data F1 score of 0.68. Finally, we tested predictions for citing networks excluding clinical citations to control for whether the prediction scores were robust due to detection of clinical trials that were already present in the data set; this resulted in a slight decrease in F1 score (from 0.68 to 0.65) but did not substantially decrease the predictive power of the model. Together, these data indicate that the features of the citing articles contribute critical information to the model’s predictive power.

To validate our machine learning approach, we compared the outputted probabilities of eventual citation by a clinical article to postpublication peer reviews. Reviewers in Hutchins and colleagues [18] were asked as part of that study to “rate the likelihood that the results (of a particular paper) could ultimately have a substantial positive impact on human health outcomes.” While this is not identical to predicting a clinical citation, if our algorithm were substantially worse at detecting translational activity than expert reviewers, this would be observable in the performance measures of accuracy, precision, and recall. We tested 985 expert reviews against machine learning predictions made with data profiles limited to only that information that was available to the experts as they completed their work in late 2014 (Fig 3C and 3D). Both the accuracy and F1 scores of algorithmically predicted clinical citations outperformed predictions based on expert ratings (81% versus 69% and 0.68 versus 0.52, respectively; *p <* 10^−6^). These results should be interpreted cautiously; the favorable results for the machine learning system do not necessarily mean that it is generally better at predicting translation than experts. Since the experts and the machine learning system are not answering exactly the same question, experts might identify translational progress not reflected in clinical citations. Nevertheless, by these limited measures, machine learning performs at least as well as expert peer-review ratings and has the advantage of being scalable to the entire PubMed database.

The surprisingly strong performance of the Random Forest model suggested that, for papers published sufficiently long ago, predicted clinical citations should correlate well with actual clinical citations. We tested this for the model in which only 2 years’ worth of data were made available in the training set. Correlation between predicted and actual citations was equally strong for papers published in 1995, 2000, and 2005 (Fig 3E). Consistent with our earlier results, using all data in place of 2 years’ worth of data did not substantially improve the correlation (S4A Fig). The machine learning model therefore appears to output accurate predictions regardless of the age of the papers.

Although our algorithm accurately predicts clinical citations, the level of uncertainty associated with the scores for individual papers is inconsistent with the precision with which the machine reports its results. We therefore binned our computationally calculated predictions into 5 stratified groups (S4B Fig), referred to as Approximate Potential to Translate (APT) scores: <5%, 25%, 50%, 75%, and >95%. Membership in any given bin is in general very stable; the correlation in article-level APT scores from the second year to the third year was 0.89, and exceeded 0.90 when comparing year 2 with later years (range 0.93–0.98). Much of the increase in APT scores resulted from articles moving out of the <5% category (Fig 3F), although movement from the other categories into the next higher one was also observed.

Two phenomena could, in principle, account for increasing prediction scores over time. The baseline odds of a paper being cited by a clinical article could have risen over time as the machine learning system detected one or more global shifts in the clinical landscape. Alternatively, the change could have been due to new, salient information entering the citing networks of the papers themselves over time as the flow of knowledge progressed. We could distinguish between these 2 possibilities by selecting articles with APT scores that increased over time, specifically those that had APT scores in the 25% category at year 2 and subsequently increased to a higher APT category (50% to 95%). We could then repeat the analysis of those papers after fixing in place either the citing network or the trained classifier, thereby testing their respective roles. If a fixed citing network produced higher scores with classifiers trained in different years, it would suggest that a shift in the baseline odds of clinical citation was responsible for the increase in APT scores. If instead the increase occurred only when the growing citing network was fed into a fixed classifier, it would suggest that additional knowledge flow—associated with the papers themselves and made possible by the passage of time—resulted in the higher likelihood of translation.

Fig 3G (blue line) shows the results for a fixed citing network; the classifiers trained in different years yielded nearly identical APT scores (the ΔAPT score hovers around 0). Conversely, APT scores increased steadily with the fixed classifier trained on growing citing networks (Fig 3G, orange line). The plateau at about year 7 agrees with the earlier suggestion that most knowledge transfer has completed by that time (see S1 Text and S4C Fig). This indicates that information in the citing networks of the papers themselves carries the increasing signatures of translation.

The receipt of clincal citations can be regarded as an early indicator of translation; however, not all clinical trials ultimately make the transition from bench to bedside. We therefore wondered how APT scores might relate to clinical trials that report positive results or those that progress to the next clinical phase. We identified 80,123 papers that were eventually cited by clinical trials meeting one or both of these criteria and analyzed their APT scores for the year before that particular clinical citation occurred, to avoid having our results confounded by our outcome measure. We found a small but statistically significant increase (approximately 5%, *p* < 10^−6^, logistic regression) in the percentage of clinical trials with either of these properties that cite an article with an APT of 95% compared to those with an APT score of 5% (S5 Fig). Although this result should be interpreted cautiously due to the small effect size, it suggests that our model is capable of detecting hallmarks of research that is more likely to have clinical impact and that APT scores may continue to convey information about translation even after a clinical citation occurs.

### Machine learning insights into the nature of knowledge flow in translational research

One general disadvantage when using machine learning for prediction is that the rules inferred by the system are not immediately obvious. The Random Forests generated here, for example, are composed of a large collection of decision trees, each with tens of thousands of nodes. However, an advantage of Random Forest machine learning models is that they allow the relative importance of various features to be calculated based on their mean decrease in Gini impurity [23] during the training stage. We were therefore able to identify the features within our data profiles that were important for determining APT scores in a random sample of 100,000 papers published over a 20-year timeframe (Table 2 and S6 Fig).

**Table 2:** Importance rank of features for prediction.

Importance rank^1^	Fundamental articles^2^^,^^3^^,^^4^	Human-focused articles^5^	All articles
1	Human (mean)	Cites/Year (P)	Cites/Year (P)
2	Human (SD)	Drug (SD)	Human (mean)
3	Cites/Year (P)	Disease (SD)	Human (SD)
4	Disease (mean)	Therap. (mean)	Disease (mean)
5	Disease (SD)	Human (mean)	Human (max)
6	Human (max)	Drug (mean)	Disease (SD)
7	Animal (SD)	Therap. (SD)	Therap. (mean)
8	Mol./Cell. (mean)	Disease (mean)	Therap. (SD)
9	Animal (mean)	Human (SD)	Drug (SD)
10	Mol./Cell. (SD)	Human (max)	Drug (mean)
11	Therap. (mean)	Animal (SD)	Mol./Cell. (mean)
12	Therap. (SD)	Animal (mean)	Animal (mean)
13	Drug (SD)	Animal (max)	Mol./Cell. (SD)
14	Drug (mean)	Mol./Cell. (SD)	Animal (SD)
15	Mol./Cell. (max)	Mol./Cell. (mean)	Human (P)
16	Animal (P)	Mol./Cell. (max)	Mol./Cell. (max)
17	Animal (max)	Therap. (P)	Animal (max)
18	Mol./Cell. (P)	Drug (P)	Mol./Cell. (P)
19	Disease (P)	Disease (P)	Animal (P)
20	Therap. (P)	Human (P)	Disease (P)
21	Drug (P)	Animal (P)	Therap. (P)
22	Human (P)	Mol./Cell. (P)	Drug (P)

^1^Relative importance of features for making predictions about eventual clinical citation.

^2^Types of MeSH terms include Human, Animal, Mol./Cell., Disease, Therap., and Drug.

^3^P indicates a property of the paper itself; all other indicators in parentheses are properties of the citing network. SD is standard deviation of the values in the citing network.

^4^Fundamental papers are those with at least one Animal or Mol./Cell. MeSH term and no Human MeSH terms.

^5^Human-focused papers are those with at least one Human MeSH term and no Animal or Mol./Cell. MeSH terms.

**Abbreviations:** Drug, Chemical/Drug; max, maximum; MeSH, Medical Subject Headings; Mol./Cell., Molecular/Cellular; Therap., Therapeutic/Diagnostic Approaches

We were most interested in discovering whether different features drove the assignment of APT scores to human-focused versus fundamental research papers, especially given the proximity of the former to clinical research (Fig 1). For fundamental papers, the mean percentage and associated standard deviation of Human MeSH terms that mapped to the citing papers were the most salient features for predicting translation; the latter measures variation within each citing network (Table 2). For human-focused papers, citation rate was the strongest predictor, followed by the degree to which modifying MeSH terms (Chemical/Drug, Disease, and Therapeutic/Diagnostic Approaches) were present in the citing network. For all papers, including the human-focused and fundamental subsets, a paper’s citation rate and the presence of Human MeSH terms in its citing network were most important for generating predictions about eventual clinical citation; other properties of the paper itself were less important than the properties of the papers that cited it. The standard deviation of features of the citing network was ranked as highly important for all papers; variation in the percentage of Human MeSH terms was ranked third most important. These importance rankings remained the same even after merging highly correlated features. Taken together, these results suggest the existence of more than one pathway of knowledge flow that moves through distinct scientific domains before reaching the clinic.

**Fig 4:**
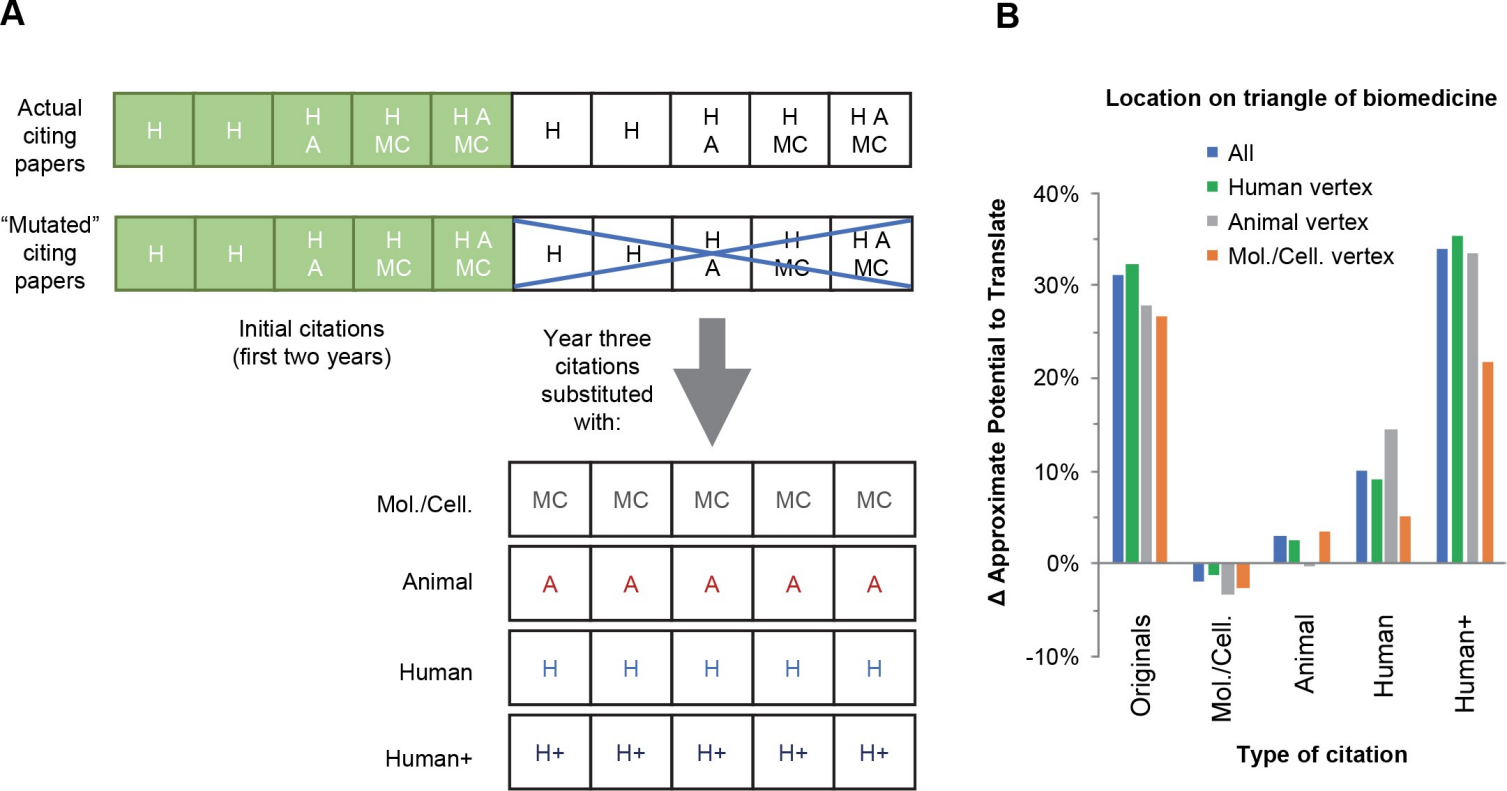
Effect on the translational potential of a paper after it receives different classes of citations. Testing the change in APT scores after directly manipulating the citing networks of 14,789 articles by adding or changing the types of citations. (A) Sample of a paper in this analysis that has 5 citations in the second year after publication (green squares) and an additional 5 citations in year 3 (white squares). We replaced the citations from year 3 with 5 citations that were MC, A, H, or H+. The same procedure was repeated for articles that populate vertices of the triangle of biomedicine (i.e., articles that were at least 95% H, A, or MC). (B) Effect on APT scores after manipulating the citing networks of all articles and for subsets articles that populate the H, A, and MC vertices. “Originals” indicates the change in APT score associated with the naturally occurring citations after year 3. A, Animal; APT, Approximate Potential to Translate; H, Human; H+, Human with Disease, Therapeutic/Diagnostic Approaches, and Chemical/Drug terms; MC, Molecular/Cellular; Mol./Cell., Molecular/Cellular.

We used 2 complementary methods in a further attempt to identify knowledge flow trajectories that culminate in successful translation. First, we applied unsupervised clustering to fundamental papers with high and low APT scores; the citing networks of those flagged as likely to translate tend to map closer to the Human vertex of the triangle of biomedicine than the networks of those unlikely to translate (S1 Text and S7 Fig). This result provides further support for the rank order of important features the machine uses to generate APT scores. Second, we analyzed the set of papers that moved into a higher APT category from one year to the next. We analyzed the 14,789 papers (3.0% of the entire test set) that had an APT score of 25% two years after publication and moved into the 50%, 75%, or >95% categories (mean APT score of 56%) after year 3 (APT↑ papers). These papers averaged a total of 5 citations in year 2 and 10 in year 3, or an increase in citations per year from 2.5 to 3.3; a representative example is shown in Fig 4A (top row).

In a proof of concept, the corresponding citing networks could be directly manipulated by adding or changing the types of citations (analogous to introducing genetic mutations and testing for gain- or loss-of-function) and re-run through the classifier to observe their new scores after manipulation (Fig 4A, second row). As a control, we tested whether simply increasing the number but not changing the type of citing papers would account for the change in APT scores. Adding duplicates of papers that were in network within the first 2 years to match the size at year 3 led to only 35% of the articles moving to a higher APT category, resulting in a mean APT score of 34% instead of 56%. Changing the size of the network was therefore insufficient to change the APT score for 65% of the papers we analyzed, suggesting that the changed characteristics of the publications in the citing network were required for the transition to a higher APT score.

We next asked how changes to the characteristics of citing networks as new papers are added would affect the assignment of APT scores by the machine (Fig 4B, blue bars). We manipulated the citing network by increasing the number of citations to equal that received by each article in year 3 and selecting papers that had specific homogeneous arrays of MeSH terms (Fig 4A, bottom). Adding Molecular/Cellular papers alone to the network actually decreased the average APT score by 2%, despite the increase in citation rate. Adding papers with solely Animal or Human MeSH terms increased the average APT score by 3% and 10%, respectively, far short of the 31% increase observed for the naturally occurring networks. In contrast, adding Human papers with Disease, Therapeutic/Diagnostic Approaches, and Chemical/Drug terms (hereafter referred to as Human+ citations) increased the average APT score to a level above the naturally occurring networks.

The results in Fig 4B and Table 2 suggested the possibility that Human+ citations play an important, perhaps even predominant, role in signaling future citation by a clinical article. We could test this for APT↑ papers by determining whether Human+ citations are overrepresented among the naturally occurring citing papers in year 3 (“Originals” in Fig 4B). This turns out to be true; in year 3, Human+ citations constitute 12.0% of all PubMed papers but 36.1% of the naturally occurring citations to APT↑ papers. However, consistent with the earlier suggestion that knowledge can reach the clinic through more than one domain, 33.4% of the APT↑ papers achieved a higher APT score without being cited by a Human+ paper.

Our results demonstrate that the way in which a paper connects to the larger biomedical research landscape, as measured by the content of different types of papers in its citing network, carries important information about its translational potential. We therefore wondered whether the HAMC content of the cited papers themselves changes the relationship between citing networks and translational potential. To test this hypothesis, we repeated our experiment with papers that fall in different locations on the trilinear graph (Fig 1), in particular those at the Human, Animal, or Molecular/Cellular vertices (Fig 4B, green, grey, and orange bars, respectively). Interestingly, the effect of the different types of citations does vary based on the content of the starting paper. For example, restricting papers located at the Animal vertex to an all Animal citing network (Fig 4B, grey bar, “Animal”) caused a small negative change in the APT score but had the opposite effect on papers located at the Molecular/Cellular vertex (Fig 4B, orange bar, “Animal”). Human citations also had variable impact, being more beneficial to papers with an Animal focus than to papers with a Molecular/Cellular focus (Fig 4B, grey and orange bars, “Human”). And consistent with the results above, reliance on Human+ citations was not universal; the latter underperformed in the naturally occurring citing networks of papers at the Molecular/Cellular vertex (Fig 4B, leftmost and rightmost orange bars). These results again demonstrated the importance of diversity in the citing network and suggest that, in normal bench-to-bedside translation, knowledge percolates through multiple domains of science before reaching clinical researchers.

## Discussion

The lengthy interval between basic research discoveries and eventual clinical applications, often measured in decades [24,25], presents significant challenges for assessing and guiding the process of bench-to-bedside translation. Few signals of success have been available at early stages of this process, when attention from researchers and/or intervention by funding agencies could have the greatest impact. By integrating information about the number and type of citations a paper receives, we demonstrate here that a machine learning system can reliably predict the successful transfer of knowledge to clinical applications. Additional research is needed to make predictions about which subset of those applications eventually impacts human health directly, most often involving the downstream generation of new drugs, devices, or improvements in clinical practice. Models that identify more granular properties of translation, such as the hallmarks of highly innovative impacts on human health (e.g., first-in-class drugs) are another potential area of future study. As machine learning and feature extraction methodologies continue to improve, it is not unreasonable to imagine that sufficiently robust data profiles can eventually be generated and used to characterize the process of translation from beginning to end and determine—for each scientific discovery—the likelihood that it will eventually lead to one or more improvements in human health.

Although our machine learning system becomes more accurate as citing networks mature and additional information becomes available, the reaction of the scientific community to a paper within the first 2 years after its publication imparts a remarkable degree of predictive power. That said, these predictions are lagging indicators in that it is necessary to wait for a more formal (literature-based) representation of the scientific community’s reaction to a paper before the likelihood that it will translate can be estimated. This is also true of our field- and time-normalized measurement of influence, the Relative Citation Ratio (RCR) [18]. Our work suggests that, in the future, it may be possible to make predictions and identify influential research by collecting less formal, closer to real-time representations of the reaction to scientific ideas and accomplishments. The overarching goal of these efforts is to provide tools that serve as an effective supplement to decision-making, both by administrators attempting to identify the most meritorious work to support and by practitioners choosing which ideas to pursue. Importantly, these tools can also look in the other direction of time’s arrow to assess past productivity of research inputs and outputs.

**Fig 5:**
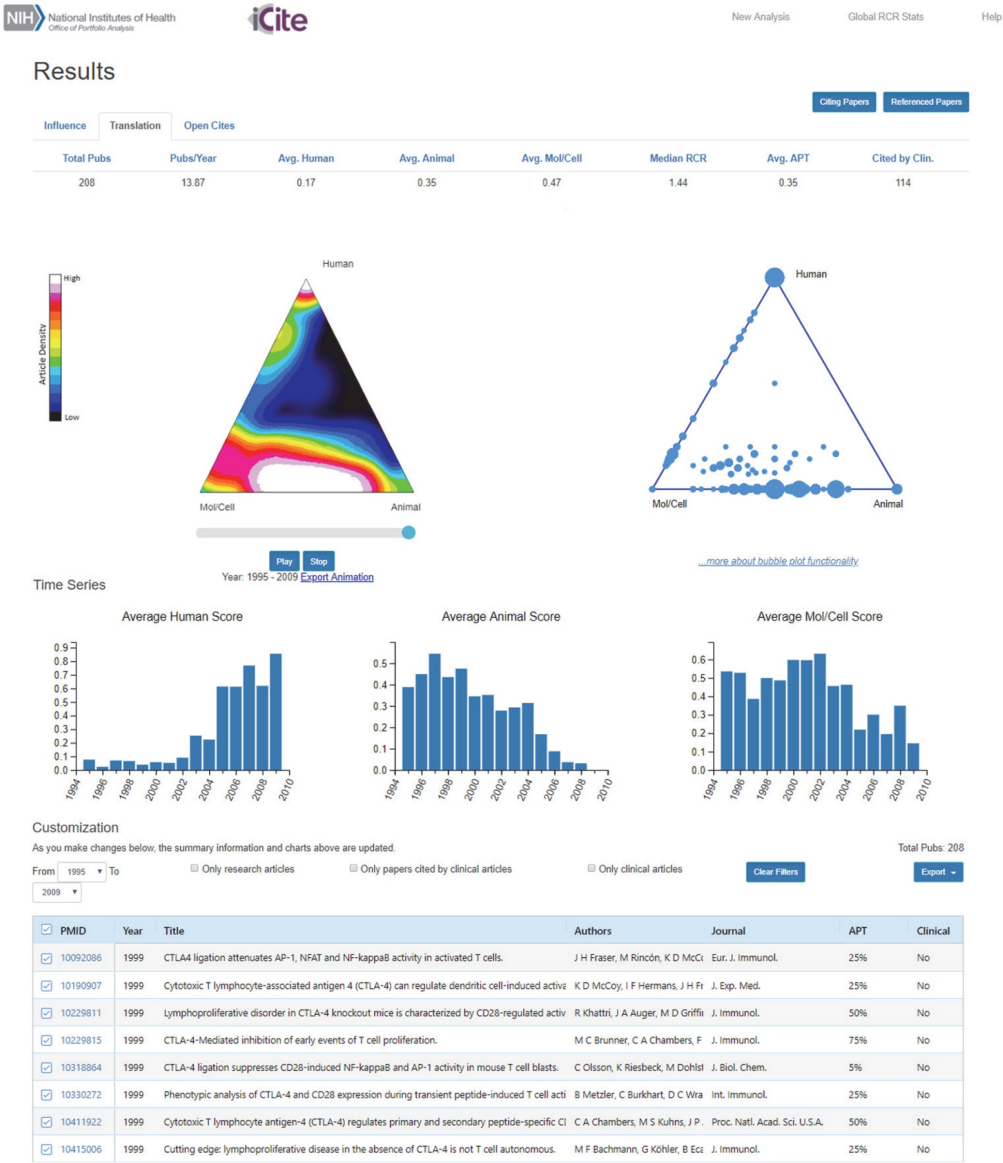
Screenshot of the translation module of *iCite*. Following standard PubMed-like searches to identify publications of interest, this new tool displays summary statistics (top) that include APT scores and the average fraction of HAMC MeSH terms, interactive trilinear graphs (middle) that include a heat map of the distribution of those publications within the triangle of biomedicine, and an interactive table (bottom) that displays the characteristics of each paper. The *iCite* tool is publicly available at https://icite.od.nih.gov/analysis. APT, Approximate Potential to Translate; HAMC, Human, Animal, and Molecular/Cellular; MeSH, Medical Subject Headings; Mol/Cell, Molecular/Cellular; RCR, Relative Citation Ratio.

Based on a prior proposal [26], a framework to independently assess Influence, Quality, Reproducibility, Sharing, and Translation (IQRST) has been suggested as a way to measure research productivity [27]. RCR-based measurement of influence, and citation of a paper by one or more clinical articles (or where that has yet to occur, its APT score), could serve as the “I” and “T” in that formula, respectively. Quality is best suited to careful evaluation by experts, a crucial step in research assessment that cannot easily be replaced [28]. Finding ways to measure the other two facets of the IQRST framework, reproducibility and data sharing, is the subject of ongoing research [29–32]. This multifaceted methodology, which eschews the reliance on any one component in favor of a more holistic approach, represents a more effective means of evaluating or comparing the impact of research portfolios [27]. Our predictions of clinical citation, which represent early signatures of bench-to-bedside translation, have been scaled to encompass the entire PubMed database; we have updated our publicly available, web-based *iCite* tool by adding a Translation module to the preexisting Influence module that has allowed users to interactively view and download RCR data. Fig 5 shows a screen capture of the *iCite* Translation module, which features a triangle of biomedicine visualizations and an option to download the underlying triangle coordinates, citation(s) by clinical articles, and APT score of each article.

Metrics used in research assessment must be made as resistant to gaming as possible. We therefore considered, as we did previously for the RCR metric [18], how an author might try to game APT scores. Perhaps the most effective strategy would be through self-citation by subsequent papers that the author crafts in a cynical attempt to add Human+ MeSH terms to the citing network. There are 3 problems with this gaming strategy: it would need to be repeated at least several times to have a chance of boosting the APT score of the cited article to a higher category; authors are not fully in control of the outcome because final assignment of MeSH terms is made by NLM indexers; and, in the absence of real value to the scientific community, the RCRs of the citing papers would remain very low. The latter consequence is one of the strengths of a multifaceted system for appraising value—gaming becomes dramatically more difficult when the target, rather than being a single variable, is a panel of metrics, each of which measures a different property. Nevertheless, we recognize the possibility that the adoption of APT scores by the scientific community may influence citation patterns and plan to reevaluate performance of the model on an annual basis.

Since APT scores appear to predict one indicator of translation at least as well as expert reviewers and are generated at scale, they may serve as a particularly valuable means of assessing the potential outcomes of fundamental research, which takes longer to be cited by a clinical article. Our results imply that an influential (high-RCR) fundamental paper will remain in the lowest (5%) APT category if its citing network remains focused on Molecular/Cellular topics, since adding those types of papers to citing networks results in a decrease in APT score. In this context it is worth revisiting the 4 seminal circadian rhythm papers cited by the 2017 Nobel Prize organization, none of which were cited by a clinical article in the 33 years following their publication and all of which have an APT score of 0.05 (as of October 1, 2018). This is an unusual case, because we estimate that the APT scores for seminal publications on research that will eventually win a Nobel prize is 2.5 times higher than that of the average paper with the same HAMC coordinates published in the same year. The papers citing the seminal research on circadian rhythms have few Human MeSH terms, plotting almost exclusively along the base of the triangle of biomedicine (S8 Fig). This is consistent with reviews of this area of research, which have concluded that more work is needed before our greatly improved fundamental understanding of the molecular mechanisms of circadian rhythms will translate into clinical applications and downstream improvements in human health [33]. The most likely way for such fundamental papers to exhibit an increase in translational potential would be citation by a group of (ideally diverse) clinical researchers. In contrast, broad scientific influence (high RCR) seems to be the largest correlate of translation for human-focused papers, since the information doesn’t have to move very far to reach clinical relevance. This, in addition to the results we obtained by manipulating citing networks, suggests that successful translation generally entails the flow of knowledge through different domains before percolating into the clinical arena.

Although this study tracked knowledge flow in translational science by using the HAMC categories and focused on citation by one or more clinical articles as an endpoint, our machine learning framework was designed to be flexible enough to tailor data profiles that enable prediction for a wide variety of biomedical research outputs and outcomes. The MeSH tree currently consists of 16 categories [34] that could be applied to a variety of distinct questions by using alternative combinations of those categories to construct the data profile inputs into our machine learning model. For example, behaviorists or social scientists might value data and visualizations based on the Disease, Behavior, and Population Characteristics branches of the MeSH ontology because those are likely to be most pertinent to their field. In principle, questions about other outcomes, such as the development of intellectual property or public health interventions, could also be predicted by using alternative data profiles.

Our findings capture translation of biomedical knowledge that is represented in the citing networks of research articles, spanning the full extent of the translational science landscape. The “gain- or loss-of-function” experiment described here, which reveals the existence of distinct channels of information flow that lead to different translational outcomes, is a proof of concept that should allow further delineation of those knowledge flow trajectories. The demonstration that scientific progress in biomedicine can be assessed and predicted in real time provides further evidence that, despite the noisiness of citation kinetics, they obey fundamental mathematical principles that lend structure to the associated dynamic networks. Although metrics alone should not replace human evaluation, these early indicators of future translation, which can be acquired during a window of opportunity compatible with shaping research strategies and portfolios, could be a productive means of accelerating progress that improves human health through better informed decision-making by investigators and funders alike.

Technical terms used throughout this article are defined in the glossary below (Box 1).

**Glossary of terms and abbreviations**

Accuracy: percent of correct predictions in the test set

APT: a machine learning-based prediction that represents the odds that a publication will eventually be cited by a clinical article. APT scores are binned into 5 groups: >95%, 75%, 50%, 25%, and <5%.

APT↑: articles that move from one APT score bin to a higher one in the subsequent year

Classifier [21]: a machine learning computational system that is trained, with input data that has been labeled as belonging to particular classes, to predict additional instances of the trained class of among previously unseen instances

F1 score [22]: harmonic mean of precision and recall

Harmonic mean: the reciprocal of the arithmetic mean of the reciprocals of a set of observations

Gini impurity [23]: a measure of the likelihood of an incorrect classification of a given variable in a classification tree if it were randomly labeled

Classification tree [35]: a type of classifier that uses decision trees to map the relationship between input observations and the associated class

HAMC: MeSH terms that fall into the Human, Animal, or Molecular/Cellular Biology categories

MeSH [20]: keywords assigned by NLM curators to describe the content of publications in PubMed, MEDLINE, and other NLM databases

RCR [18]: an article-level metric of scientific influence that adjusts a paper’s citation rate to account for field and year of publication

## Methods

### Article classification

Articles are classified as Human, Animal, or Molecular/Cellular Biology, or a combination of these three, according to Weber’s algorithm for using the MeSH terms [4], which are assigned to articles by NLM indexers [36]. MeSH terms persist once assigned, although additional terms may be added over time as the MeSH vocabulary expands. Article data were downloaded in XML format from the NLM (2016 baseline from [37]); articles in this data set were old enough to have been assigned MeSH terms at the time of download. Citation data were acquired from the database underpinning *iCite* [18,38]; except for the data related to the seminal publications that led to Nobel Prizes (Table 1; data downloaded October 2018), all results are based on a data set that was extracted in March 2016. MeSH terms were extracted from the XML for each article, and each MeSH term was mapped to its address or addresses on the MeSH tree [20]. Terms with addresses corresponding to the address B01.050.150.900.649.801.400.112.400.400 (Humans) or beginning with M01 (Persons) were classified as Human. Terms with an address beginning with B01 (Eukaryota), except the address corresponding to Humans, were classified as Animal. Terms with an address beginning with A11 (Cells), B02 (Archaea), Bacteria (B03), Viruses (B04), G02.111.570 (Molecular Structure), or G02.149 (Chemical Processes) were classified as Molecular/Cellular Biology. Note that the address for Chemical Processes has moved in the current MeSH tree, but the data set used in this paper is unaffected by this change. Articles could have more than one of these 3 classifications, or none of these.

The following PubMed queries were used to identify clinical articles:

Clinical trials:

(("clinical trial"[Publication Type] OR "clinical trial, phase i"[Publication Type] OR "clinical trial, phase ii"[Publication Type] OR "clinical trial, phase iii"[Publication Type] OR "clinical trial, phase iv"[Publication Type]) OR "clinical study"[Publication Type]).

Guidelines:

"guideline"[Publication Type]

### Trilinear data visualization

To visualize three-dimensional HAMC scores on a two-dimensional plot, these coordinates were converted to X-Y coordinates with the following equations for visualizing trilinear graphs [4]:

$$x=H\;\times 0+A\;\times \;\frac{\sqrt{3}}{2}+MC\;\times \;\frac{-\sqrt{3}}{2}$$

y=H×1+A×−0.5+MC×−0.5

The constants in these equations correspond to the x and y coordinates of the equilateral triangle vertices: (0, 1) for Human, $\left(\frac{\sqrt{3}}{2},\;-0.5\right)$ for Animal, and $\left(-\frac{\sqrt{3}}{2},\;-0.5\right)$ for Molecular/Cellular Biology.

To visualize graphs as article density plot, each article was plotted in ImageJ as a point light source at its calculated X-Y coordinates, which could be identical for each paper. Articles with identical coordinates were given a weight of 1 and summed at the overlapping point (e.g., if 10 articles appeared at the Animal axis, that point would have a value of 10). A gaussian blur function was applied in ImageJ, and the image pixel values were pseudocolored according to their resulting density value.

### Machine learning framework and validation

To build a vector space for machine learning, the following information about a research article’s properties and the properties of the set of articles that had cited it were aggregated.

The properties of the article were as follows:

H: Human score of the article

A: Animal score of the article

MC: Molecular/Cellular score of the article

D: Binary flag (0 or 1) indicating whether a Disease MeSH term was present (the “C” branch of the MeSH tree, except C22, Animal Diseases)

E: Binary flag (0 or 1) indicating whether a Therapeutic/Diagnostic Approaches MeSH term was present (the “E” branch of the MeSH tree, except E07, Equipment and Supplies)

CD: Binary flag (0 or 1) indicating whether a Chemical/Drug MeSH term was present (the “D” branch of the MeSH tree)

CPY: Article citation rate

The properties of the set of citing articles were as follows:

- maxH, meanH, sdH: Maximum, mean, and standard deviation of Human scores of the set of citing articles
- maxA, meanA, sdA: Maximum, mean, and standard deviation of Animal scores of the set of citing articles
- maxMC, meanMC, sdMC: Maximum, mean, and standard deviation of Molecular/Cellular Biology scores of the set of citing articles
- meanD, sdD: Mean and standard deviation of the Disease scores (D, as described above) of the set of citing articles
- meanE, sdE: Mean and standard deviation of the Therapeutic/Diagnostic Approaches scores (E, as described above) of the set of citing articles
- meanCD, sdCD: Mean and standard deviation of the Chemical/Drug scores (CD, as described above) of the set of citing articles

Outcomes supplied to the machine learning framework were binary flags (0 or 1) indicating whether the article had been cited by a clinical trial or clinical guideline. For some experiments, the citing network was limited to information that would have been available at a certain time (i.e., 2 years after publication), with outcomes measured in a specified time window that did not overlap with the time window in the citing network used to construct the vector space (years 3–10).

To select a machine learning algorithm, we tested Random Forests [21], Support Vector Machines [39], logistic regression [40], Maxent [41], LibLinear [42], and Neural Networks [43,44]. Random Forests consistently had the best performance, so this algorithm was selected. To validate performance, mean accuracy (i.e., percent correct predictions) and F1 scores are reported using 10 independent train/test sets drawn from 9,001,101 papers published from 1994 to 2009. Random Forests were trained with 100,000 articles and validated against 100,000 out-of-sample articles held out of the training set, with 2-fold downsampling of the more common class (papers not cited by clinical articles) during training.

The Random Forest output takes the form of the percent of decision trees “voting” that an article belongs to the positive class (i.e., cited by a clinical article), so the raw prediction output is not guaranteed to be linearly related to the actual percentages of articles in that class (although there should be a strong relationship between the predicted and actual distributions). To eliminate any nonlinearities in the raw Random Forest output, predictions were binned into 5 contiguous groups centered around the actual distribution of positive cases in each bin: <5%, 25%, 50%, 75%, and >95% odds of clinical citation. These 5 bins constitute the APT score. For time series analysis of APT scores and clinical citation, a set of 500,787 papers published in 2000 was analyzed using historical citation data that would have been available at the end of each year (2000 to 2014).

To validate machine learning accuracy, we compared the predictive power of APT scores versus expert review scores on clinical citation. We used postpublication peer reviews conducted by Hutchins and colleagues [18]; reviewers were asked to “rate the likelihood that the results could ultimately have a substantial positive impact on human health outcomes.” In order to align the information available to the reviewers at the time of the reviews with the information available to the machine learning system, we trained classifiers on citation data through the end of 2014, when the reviews were conducted. APT scores were generated for the same papers that were reviewed (985 reviews in total). These 2 sets of scores were used as predictors. Five thresholds were used for each set of scores, the supplied scores from reviewers (1–5) and the 5 APT score bins (<5%, 25%, 50%, 75%, and >95%), and receiver operating characteristic curves and precision-recall graphs were generated. Area under the curve (AUC) was measured alongside maximum F1 scores and maximum accuracy for each set of scores.

### Properties of clinical trials with downstream indicators of clinical relevance

To analyze whether APT scores convey additional information about translational success, we examined clinical trials with 2 downstream outcomes that are indicators of clinical relevance: positive results and progression to the next phase of trial. For each of these measures, an appropriate 5-year window was chosen, and publications receiving a clinical citation that could be matched with one of these outcomes were included.

For positive results, papers that were cited by a publication from 2010 to 2015 that was (a) flagged as clinical according to the above criteria, (b) could be matched to a registered clinical trial in clinicaltrials.gov that reported results and significance values for the primary outcome, and (c) was a Superiority type trial to ease interpretation of the statistical results. This time window was chosen to maximize the number of trials that had improved reporting of data after the FDA Amendments Act of 2007 expanded the clinical trials registry databank. Outcomes were considered positive if the matched, citing clinical trial reported statistically significant results for the primary outcome measure and negative if it reported nonstatistically significant results.

For progression, papers that were cited by a publication from 1995 to 2000 that was (a) flagged as clinical according to the above criteria and (b) was identified as a Phase I–III trial were included. This time window was chosen to allow time for subsequent trials to be performed and published. Outcomes were considered positive if the citing Phase I–III clinical trial was subsequently cited by a trial one phase higher, and negative otherwise.

For both outcomes, in order to avoid properties of the trial in question from being reflected in the APT score, historical APT scores from the year preceding this clinical citation were used. Logistic regression was performed controlling for the year of the trial, for whether a different clinical article had previously appeared in the citation network (e.g., clinical guidelines, trials that did not report their phase in PubMed, trials that lacked a corresponding clinicaltrials.gov entry, or trials in clinicaltrials.gov that did not report statistical results), and—for the progression outcome—the phase of the citing trial in question.

### Citation editing

A subset of the 500,787 articles published in 2000, those that had a 25% APT score in 2002 and a 50% or higher APT score in 2003, were selected for analysis (14,789 articles met these criteria). To evaluate the effects of the characteristics of the growing citing network on this observed increase in APT scores, citations from 2003 were deleted and replaced with an equal number of artificial citations that had different characteristics:

1. Originals: An equal number of articles as the deleted citations from 2003 but randomly sampled from the 2002 citing network. This ensured that the characteristics of the 2003 citing network mirrored the 2002 network, but with the higher average citation rate of the 2003 network.
2. Molecular/Cellular: An equal number of articles as the deleted citations from 2003 but with Molecular/Cellular Biology scores equal to 100% and all others equal to 0%.
3. Animal: An equal number of articles as the deleted citations from 2003 but with Animal scores equal to 100% and all others equal to 0%.
4. Human: An equal number of articles as the deleted citations from 2003 but with Human scores equal to 100% and all others equal to 0%.
5. Human+: An equal number of articles as the deleted citations from 2003 but with Human scores, Disease scores, Therapeutic/Diagnostic Approaches scores, and Chemical/Drug scores equal to 100% and all others equal to 0%.

In one experiment, all of the citing articles were deleted from the network and replaced with a homogenous set of artificial articles with Human scores equal to 100% and all others equal to 0%.

This same procedure was repeated for articles that reside in the HAMC vertices of the triangle of biomedicine (at least 95% of MeSH terms in these articles were Human, Animal, or Molecular/Cellular), to test the possibility of heterogeneity in the effect of changing citing networks of subsets of articles.

### Data availability

Data and underlying citations from the NIH Open Citation Collection [45] can be retrieved from the *iCite* web service at https://icite.od.nih.gov, through the *iCite* API at https://icite.od.nih.gov/api, or in bulk downloads from https://doi.org/10.35092/yhjc.c.4586573.

## Supporting information

S1 Text Supporting text. (DOCX)

S1 Movie Key papers contributing over time to cancer immunotherapies such as nivolumab (Opdivo), progressing from fundamental research early on (bottom) to translational (middle) and clinical studies (top). Same information and pseudocolor scale as S2D–S2F Fig.
(AVI)

S2 Movie Density graph showing the fraction of papers that have been cited by a clinical article, at each point along the trilinear graph over time. Same information and pseudocolor scale as Fig 1D–1F.
(AVI)

S1 Fig Properties of papers published in 1995 that are or are not cited by a clinical article. (A) Total citations accrued between 1995 and 2015 for papers published in 1995 that have received a clinical citation (blue), have never received a clinical citation (orange), are human focused (grey), or focus on fundamental research (yellow). The line in each box represents the median, and the x represents the mean citations; outliers were excluded. (B) Word cloud of 1,000 randomly selected 1995 papers that accrued only 8 total citations but were nevertheless cited by one or more clinical articles.
(TIF)

S2 Fig Fractional counting improves assignment of article coordinates. (A, B) Fractional counting compared to binary and manually curated article coordinates for human embryonic kidney cell research (panel A) and humanized mouse model research (panel B). Manually curated coordinates were compared with 2 algorithms for counting MeSH terms: binary counting (yes/no) and fractional counting (percentage of total). Points represent the mean coordinates of over 50 papers in each group. (C) Timeline of research on Bruton’s tyrosine kinase in cancer treatment, using fractional counting. (D–F) Cumulative density graphs of fundamental (panel D), translational (panel E), and clinical (panel F) research that led to breakthrough cancer immunotherapy treatments such as nivolumab (Opdivo).
(TIF)

S3 Fig Density graph of the percentage of papers across the trilinear graph cited by clinical articles 20 years after publication, excluding publications with only one MeSH term. (TIF)

S4 Fig Validation and stability of machine learning predictions. (A) Correlation between predicted and actual citations for papers published in 1995, 2000, and 2005, using all available citing network data for predictions. Dashed lines represent a linear regression; the slope and r^2^ values, respectively, for each year are as follows: 1995 (0.957, 0.996); 2000 (0.957, 0.989); and 2005 (0.903, 0.976). (B) Raw predictions compared to APT score assignment. (C) Average APT score for a set of articles published in the same year increases over time as citing networks mature.
(TIF)

S5 Fig Percentage of clinical trials citing an article of a given APT score that (A) report positive results or (B) progress to the next clinical phase. Data for clinical trials that reported results were retrieved from clinicaltrials.gov and normalized to the percentage of clinical trials overall that meet these criteria. Both sets of results are statistically significant (p < 10^−6^): for (A), statistical significance was calculated with a logistic regression controlling for the year of the trial and whether a different clinical article was previously present in the citation network of the article of interest, and for (B), a logistic regression controlling for the phase of the clinical trial, the year of the trial, and whether a different clinical article was previously present in the citation network of the article of interest was used.
(TIF)

S6 Fig Quantification of relative importance of features for prediction. (A) Fundamental articles, (B) Human-focused articles, (C) all articles. Black bars: citations per year; blue bars: network features; orange bars: paper features. P indicates a property of the paper itself; all other indicators in parentheses are properties of the citing network. SD is standard deviation of the values in the citing network. Values were based the mean decrease in Gini impurity for each feature during the training stage.
(TIF)

S7 Fig Hierarchical clustering to identify common knowledge flow trajectories that are linked to high or low odds of translational success. (A) Heatmap of the distance matrix for a random sample of fundamental papers from the 5% APT score and 75% APT score categories, according to the similarity of their article and citing network properties, arranged by hierarchical clustering. Papers from these 2 APT score bins appeared in the hierarchical clustering as discrete groups of consecutive, similar papers (APT score in blue on the x- and y-axes of the similarity matrix). These groups constitute common knowledge flow trajectories that have different measurable outcomes (APT score) with respect to clinical translation. Four groups are bracketed and their properties visualized. (B, C) Two sets of clusters from unsupervised hierarchical clustering are composed of similar articles (panel B, predominantly Animal-focused papers; panel C, predominantly Molecular/Cellular and Animal-focused papers) but very different APT scores. In both cases, the papers in the 75% APT score cluster are cited by papers that are more human-focused than their counterparts in the 5% APT score cluster.
(TIF)

S8 Fig Citing network of seminal publications leading to the 2017 Nobel Prize in Physiology or Medicine, plotted on the triangle of biomedicine. Density graph of HAMC coordinates for the 270 publications citing at least one of the 4 seminal papers leading to the 2017 Nobel Prize for Physiology or Medicine on the topic of circadian rhythms (PMIDs: 16593450, 6435882, 6094014, and 6440029).
(TIF)

## Abbreviations

APT: Approximate Potential to Translate
AUC: area under the curve
FDA: Food and Drug Administration
HAMC: Human, Animal, and Molecular/Cellular
MeSH: Medical Subject Headings
NLM: National Library of Medicine
PMID: PubMed Identifier
RCR: Relative Citation Ratio
