# Supplementary material for: Predicting translational progress in biomedical research
Source: PLoS Biol. 2019 Oct 10;17(10):e3000416. doi: 10.1371/journal.pbio.3000416 (PMC6786525; doi:10.1371/journal.pbio.3000416)
Supplement: S1 Text — (DOCX) [file pbio.3000416.s001.docx]

**Supporting Information**

**Supplemental Text**

***Fractional counting improves article classification***

To maximize the utility of Griffin Weber’s research classifications [1] and more accurately quantify knowledge flow along the Human, Animal, and Molecular/Cellular (HAMC) axes, we improved resolution of article mapping on the trilinear graph using fractional counting of Medical Subject Heading (MeSH) terms. The original algorithm is restricted to seven discrete points on the trilinear graph: the three vertices, the midpoints of the axes, and the center of the graph. It is also possible for an article to be off the graph, if it lacks any HAMC MeSH terms. Using the original algorithm with binary counts, an article with a total of 12 MeSH terms (6 Human, 3 Animal, and 3 Mol./Cell.), would be located at the center of the triangle. Using fractional counting, it would be scored as 50% Human, 25% Animal, and 25% Molecular/Cellular Biology, and would be shifted closer to the Human vertex than to either the Animal or Molecular/Cellular vertices (for additional examples and visualization, see Fig 1C).

We verified that fractional counting does not introduce unwanted systematic error into the article coordinates and that the method is effective for groups of articles by comparing the accuracy of the two counting approaches against expert-curated coordinates for two sets of articles: research involving Human Embryonic Kidney 293 cells, which are a commonly used cell line in cellular and molecular biology experiments, and humanized mouse models, in which human genes have been introduced to mouse models (S2A, B Fig). Fractional counting, which we applied to more than 50 articles in each case, improved performance, bringing algorithmically-generated coordinates into closer alignment with expert-curated coordinates.

Using fractional counting is also advantageous in that it opens up the full space of the trilinear graph, allowing rich visualizations of article portfolios of various sizes. We can use these visualizations to track the timeline of research from bench to bedside. S2C Fig shows the timeline for a set of articles that contributed to the development of ibrutinib, which is used to treat chronic lymphocytic leukemia by downregulating the Bruton’s tyrosine kinase signaling pathway. The work began with fundamental research on kinase signaling mechanisms in the late 1990s [2] and culminated in clinical trials in the 2010s [3,4]. For larger portfolios, fractional counting enables density graphs to be constructed from the article-level data; this kind of graph cannot be generated using binary flags without aggregating articles in some way. S2D-F Fig and S1 Movie show the density graph of a network of papers on cancer immunotherapy, including the fundamental, translational, and clinical work that eventually led to the approval of cancer immunotherapy drugs like Opdivo (nivolumab) over time, from the late 1980s to the late 2010s. Fractional counting of MeSH terms therefore has several advantages overall: it improves accuracy of article classification and enables computational approaches for visualizations that scale across any size research portfolio, while minimizing bias that can be introduced by manual curation.

***APT score stability***

Because citing networks mature as papers age and more information becomes available, it was important to ask how these machine learning predictions change over time. To test the stability of APT scores, we generated historical datasets of those predictions by limiting the data to only what was available a specific number of years beyond the publication date. This was done for articles published in the same year and with citations extending through the end of the year in each of the ensuing 15 years. Average APT scores rise over the first few years as citing networks grow and more information becomes available, and plateau at about year seven (S4C Fig), suggesting that most knowledge transfer has completed by that time, even for papers that had yet to be cited by one or more clinical articles.

***Hierarchical clustering to identify common knowledge flow trajectories***

We tested whether we could observe common knowledge flow trajectories of successful translation by analyzing the results of unsupervised clustering of articles with high and low APT scores. We focused on fundamental articles (those with 0% Human MeSH terms) to gain insights about translation of fundamental research. First, we built a similarity matrix comparing 500 randomly selected low-APT score papers (5%) and 500 high-APT score papers (75%); these bins are maximally distant for fundamental publications. Since high citation rates are correlated with translation, that variable was excluded from the similarity calculations (i.e. the clustering algorithm was blinded to the APT scores). This focused the unsupervised clustering on the direction rather than the magnitude of knowledge flow.

Hierarchical clustering revealed that the clusters are predominantly comprised of articles with either low or high APT scores (S7A Fig). We generated trilinear density graphs of the articles in these clusters (S7B, C Fig; the top row of density graphs represents the mean location of the citing articles and the bottom row represents the locations of the articles themselves). Two of the clusters (S7B Fig) are comprised of articles near the Animal vertex but have different citing networks. The articles in Cluster 1 have low APT scores (<5%) and were cited predominantly by papers with a high percentage of Animal MeSH terms, whereas the articles in Cluster 4 had high APT scores (>75%) and were predominantly cited by papers with a mixture of Human and Animal MeSH terms. We observed a similar pattern with Clusters 2 and 3 (S6C Fig), which were both predominantly comprised of articles that mapped between the Mol./Cell. and Animal vertices. Taken together, these results suggest that distinct knowledge flow trajectories are required for successful translation; for fundamental research those trajectories transit telltale signposts of increased scientific diversity and focus on humans.

**Supplemental References**

1. Weber GM. Identifying translational science within the triangle of biomedicine. Journal of translational medicine. 2013;11:126. PMID: 23705970

2. Craxton A, Jiang A, Kurosaki T, Clark EA. Syk and Bruton's tyrosine kinase are required for B cell antigen receptor-mediated activation of the kinase Akt. J Biol Chem. 1999;274(43):30644-50. PMID: 10521450

3. Burger JA, Tedeschi A, Barr PM, Robak T, Owen C, Ghia P, et al. Ibrutinib as Initial Therapy for Patients with Chronic Lymphocytic Leukemia. N Engl J Med. 2015;373(25):2425-37. PMID: 26639149

4. Byrd JC, Furman RR, Coutre SE, Flinn IW, Burger JA, Blum KA, et al. Targeting BTK with ibrutinib in relapsed chronic lymphocytic leukemia. N Engl J Med. 2013;369(1):32-42. doi: 10.1056/NEJMoa1215637. PMID: 23782158
