# Supplementary figures and images for: Predicting translational progress in biomedical research
Source: PLoS Biol. 2019 Oct 10;17(10):e3000416. doi: 10.1371/journal.pbio.3000416 (PMC6786525; doi:10.1371/journal.pbio.3000416)

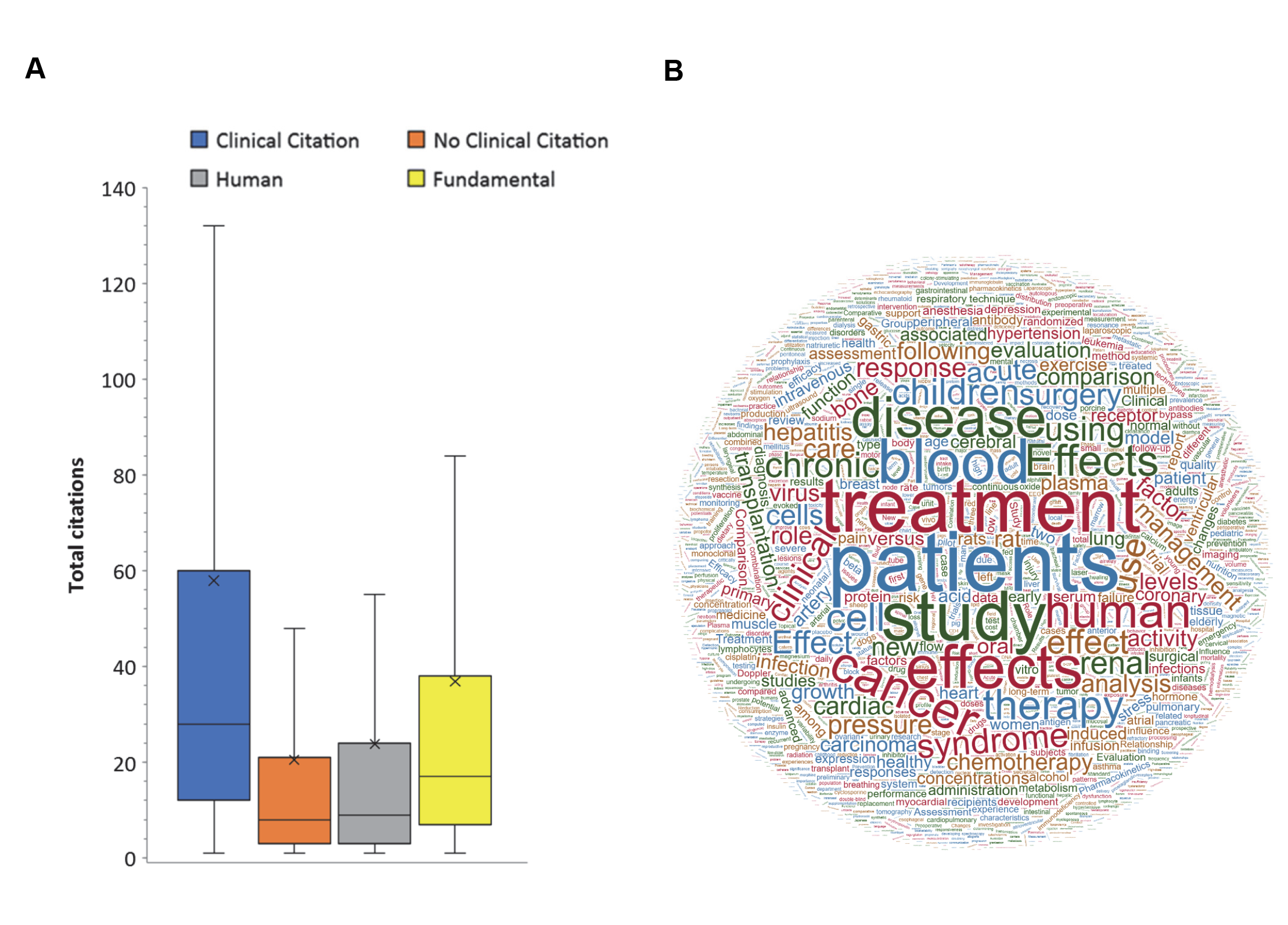

Supplement: S1 Fig — (A) Total citations accrued between 1995 and 2015 for papers published in 1995 that have received a clinical citation (blue), have never received a clinical citation (orange), are human focused (grey), or focus on fundamental research (yellow). The line in each box represents the median, and the x represents the mean citations; outliers were excluded. (B) Word cloud of 1,000 randomly selected 1995 papers that accrued only 8 total citations but were nevertheless cited by one or more clinical articles. (TIF) [file pbio.3000416.s004.tif]

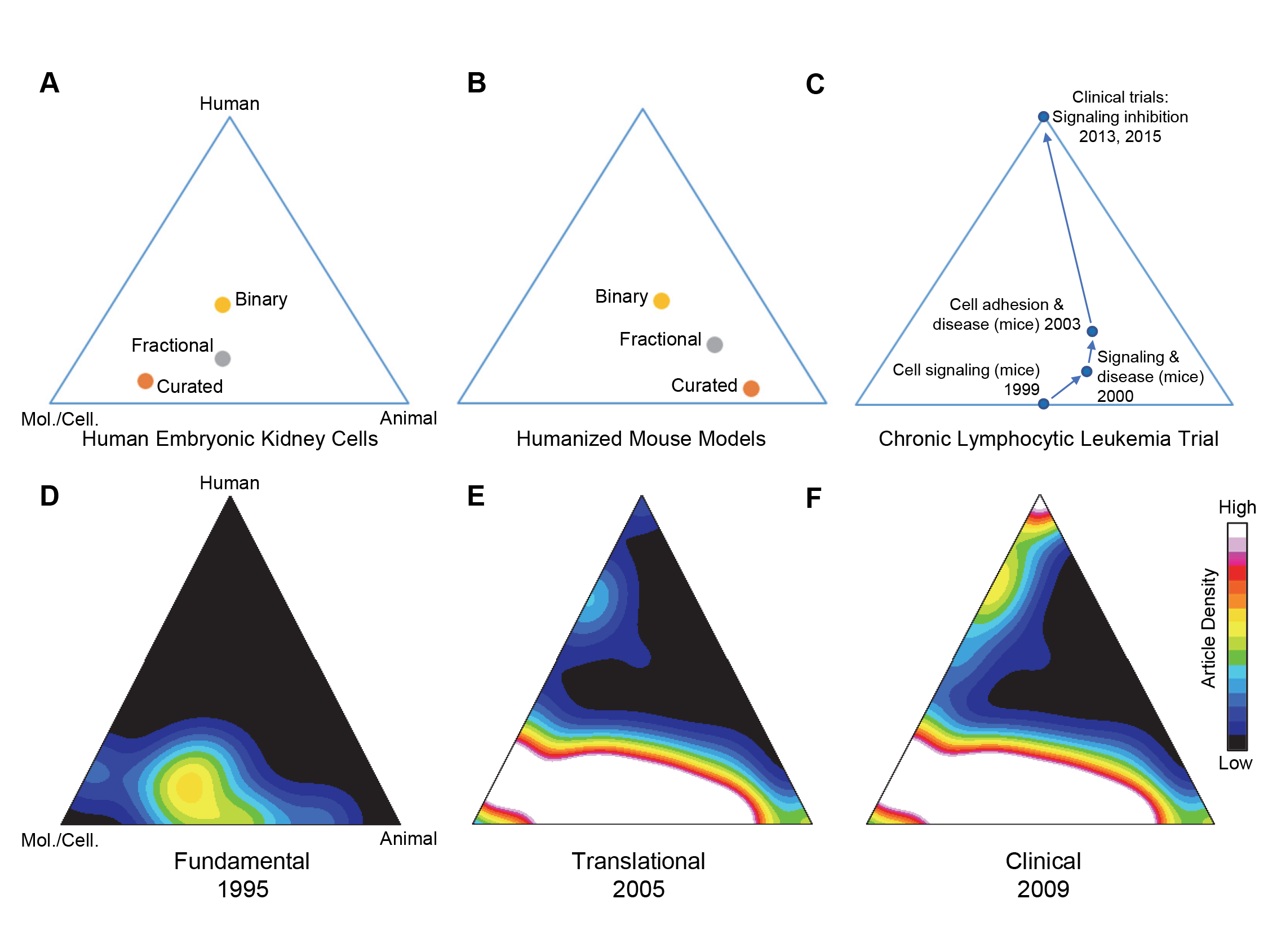

Supplement: S2 Fig — (A, B) Fractional counting compared to binary and manually curated article coordinates for human embryonic kidney cell research (panel A) and humanized mouse model research (panel B). Manually curated coordinates were compared with 2 algorithms for counting MeSH terms: binary counting (yes/no) and fractional counting (percentage of total). Points represent the mean coordinates of over 50 papers in each group. (C) Timeline of research on Bruton’s tyrosine kinase in cancer treatment, using fractional counting. (D–F) Cumulative density graphs of fundamental (panel D), translational (panel E), and clinical (panel F) research that led to breakthrough cancer immunotherapy treatments such as nivolumab (Opdivo). (TIF) [file pbio.3000416.s005.tif]

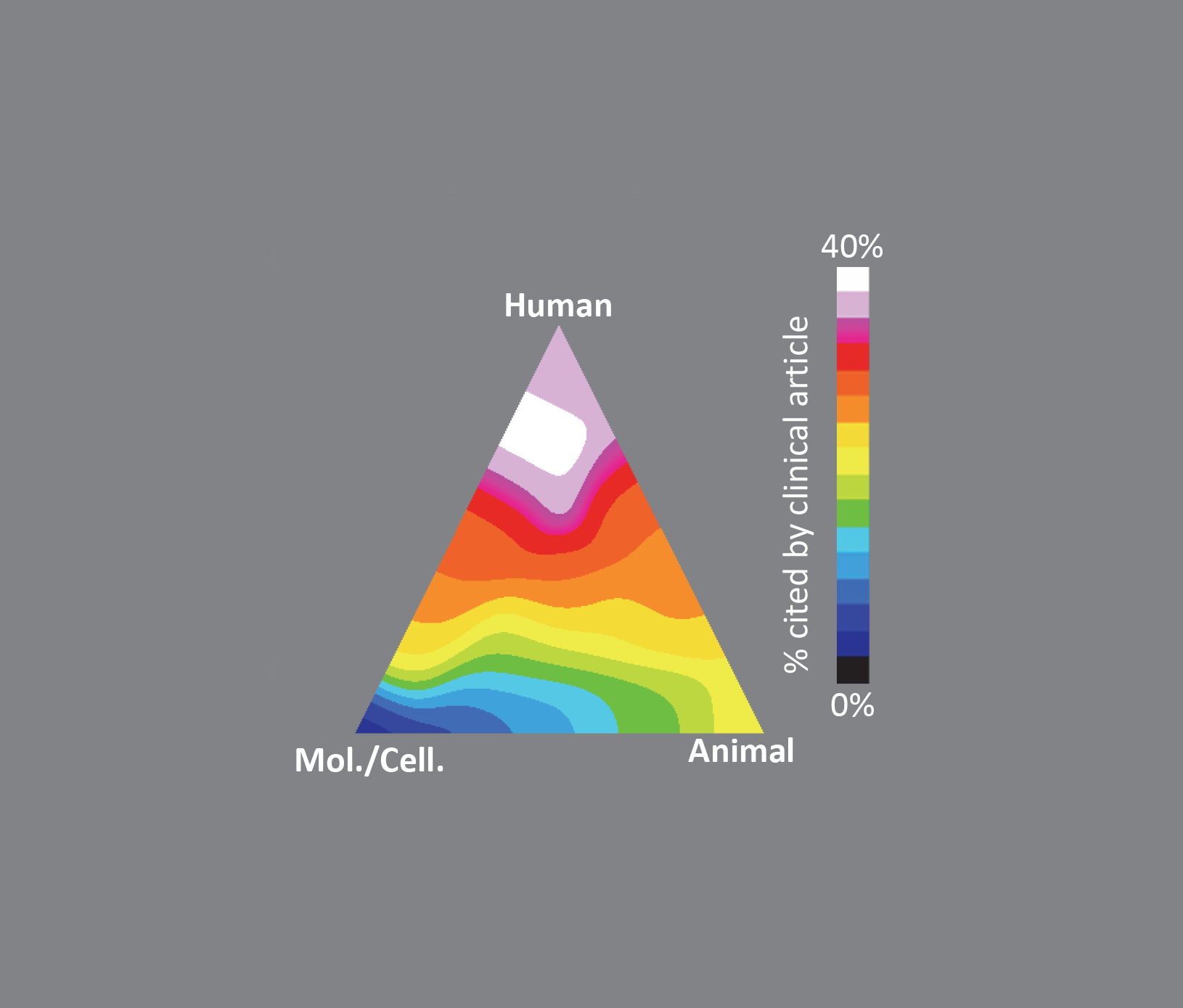

Supplement: S3 Fig — (TIF) [file pbio.3000416.s006.tif]

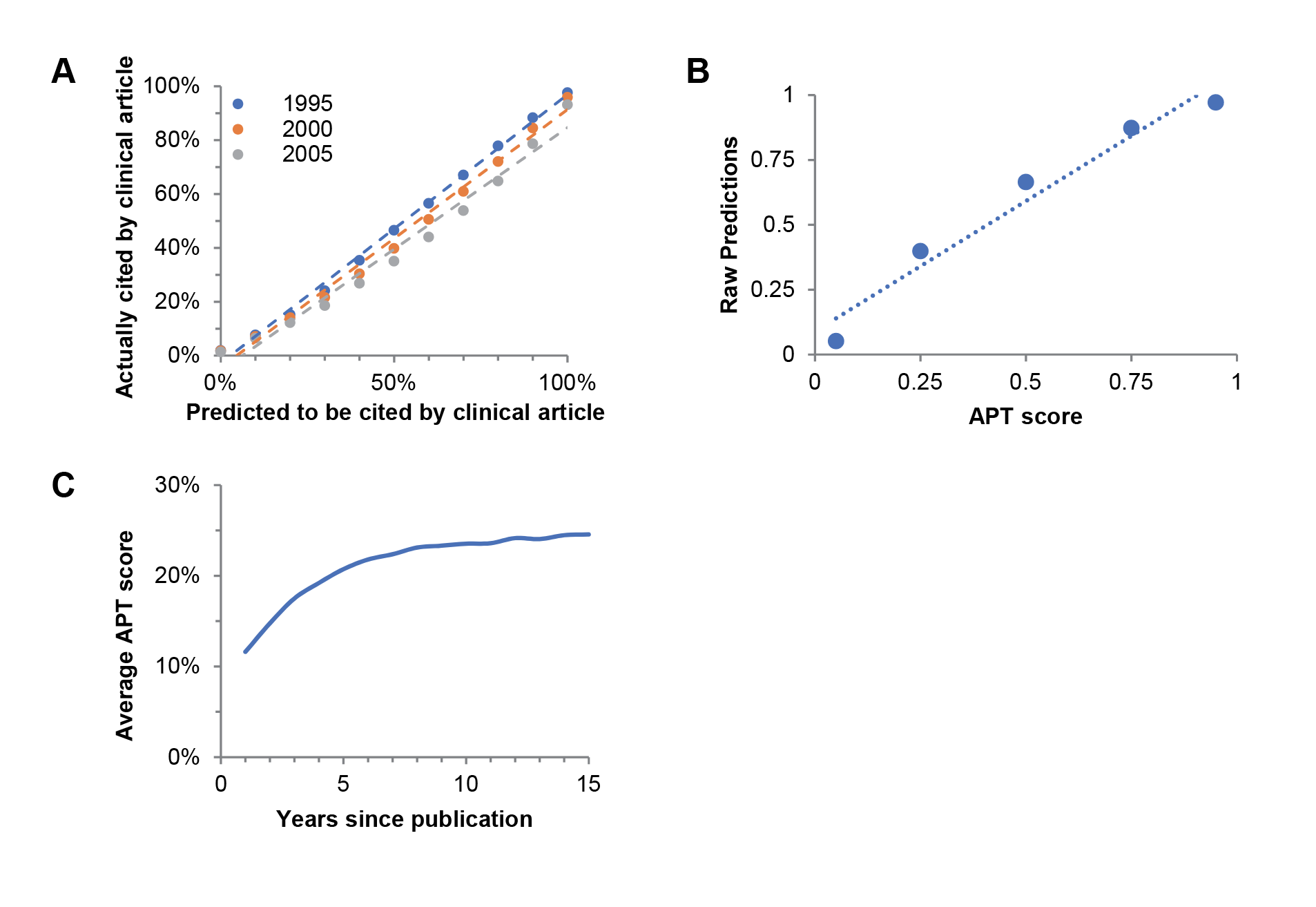

Supplement: S4 Fig — (A) Correlation between predicted and actual citations for papers published in 1995, 2000, and 2005, using all available citing network data for predictions. Dashed lines represent a linear regression; the slope and r2 values, respectively, for each year are as follows: 1995 (0.957, 0.996); 2000 (0.957, 0.989); and 2005 (0.903, 0.976). (B) Raw predictions compared to APT score assignment. (C) Average APT score for a set of articles published in the same year increases over time as citing networks mature. (TIF) [file pbio.3000416.s007.tif]

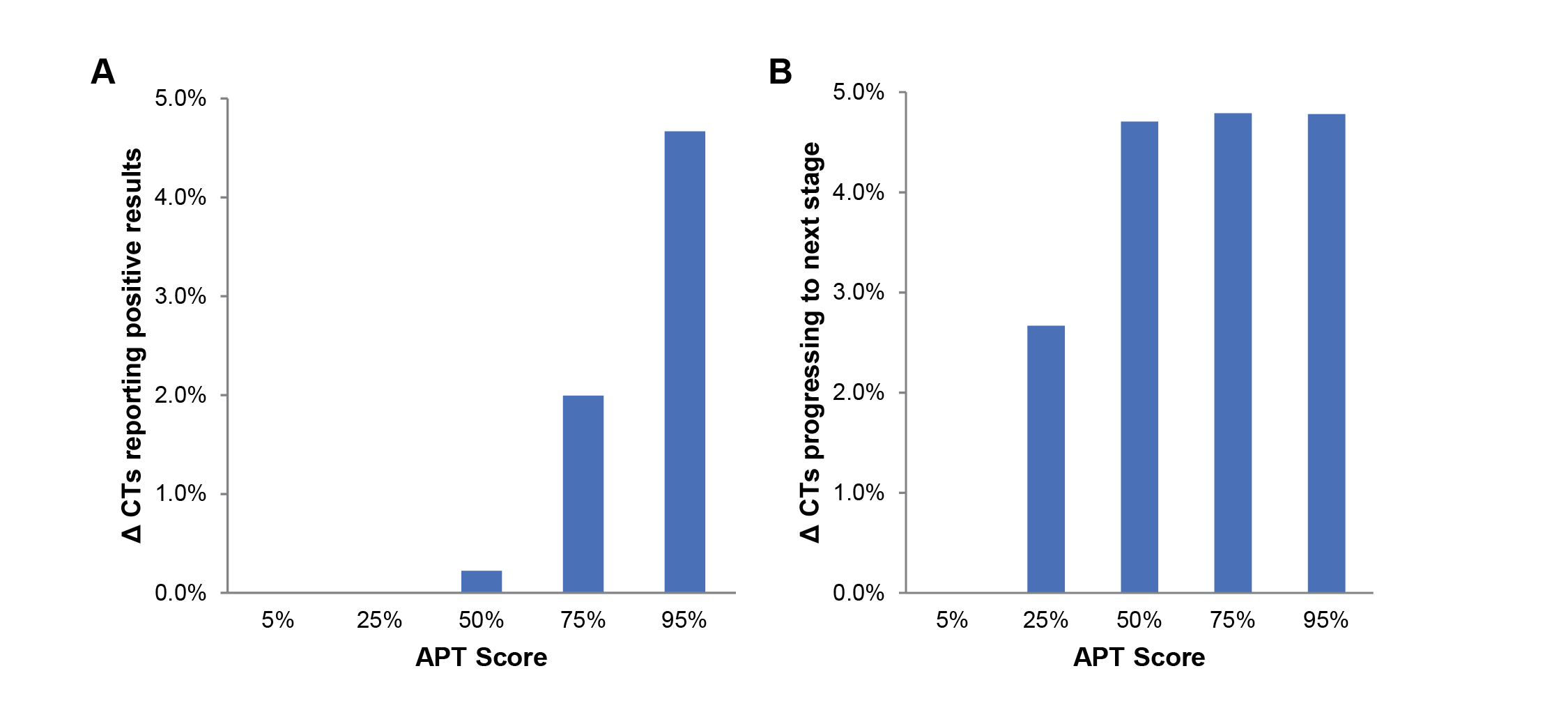

Supplement: S5 Fig — Percentage of clinical trials citing an article of a given APT score that (A) report positive results or (B) progress to the next clinical phase. Data for clinical trials that reported results were retrieved from clinicaltrials.gov and normalized to the percentage of clinical trials overall that meet these criteria. Both sets of results are statistically significant (p < 10−6): for (A), statistical significance was calculated with a logistic regression controlling for the year of the trial and whether a different clinical article was previously present in the citation network of the article of interest, and for (B), a logistic regression controlling for the phase of the clinical trial, the year of the trial, and whether a different clinical article was previously present in the citation network of the article of interest was used. (TIF) [file pbio.3000416.s008.tif]

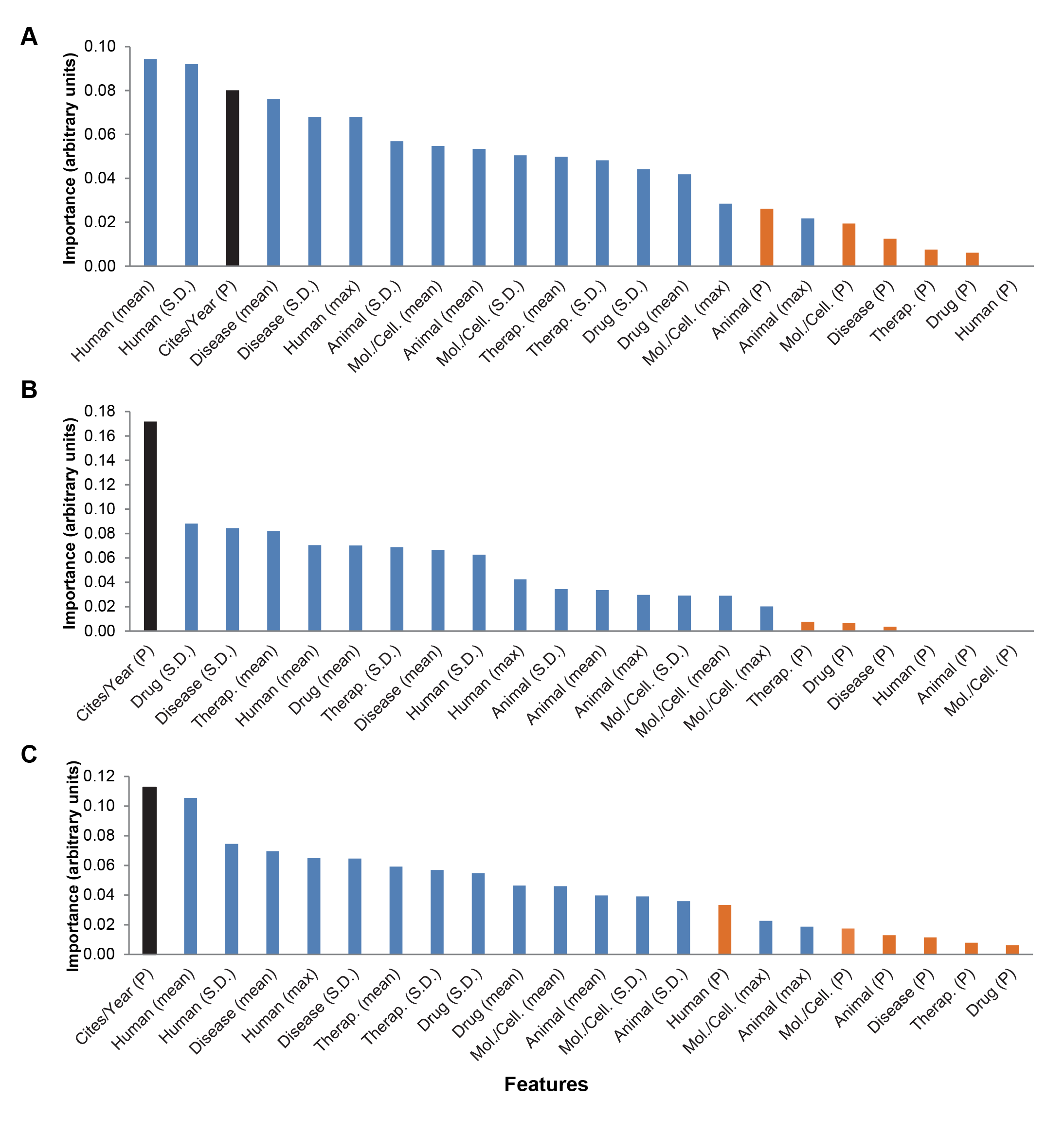

Supplement: S6 Fig — (A) Fundamental articles, (B) Human-focused articles, (C) all articles. Black bars: citations per year; blue bars: network features; orange bars: paper features. P indicates a property of the paper itself; all other indicators in parentheses are properties of the citing network. SD is standard deviation of the values in the citing network. Values were based the mean decrease in Gini impurity for each feature during the training stage. (TIF) [file pbio.3000416.s009.tif]

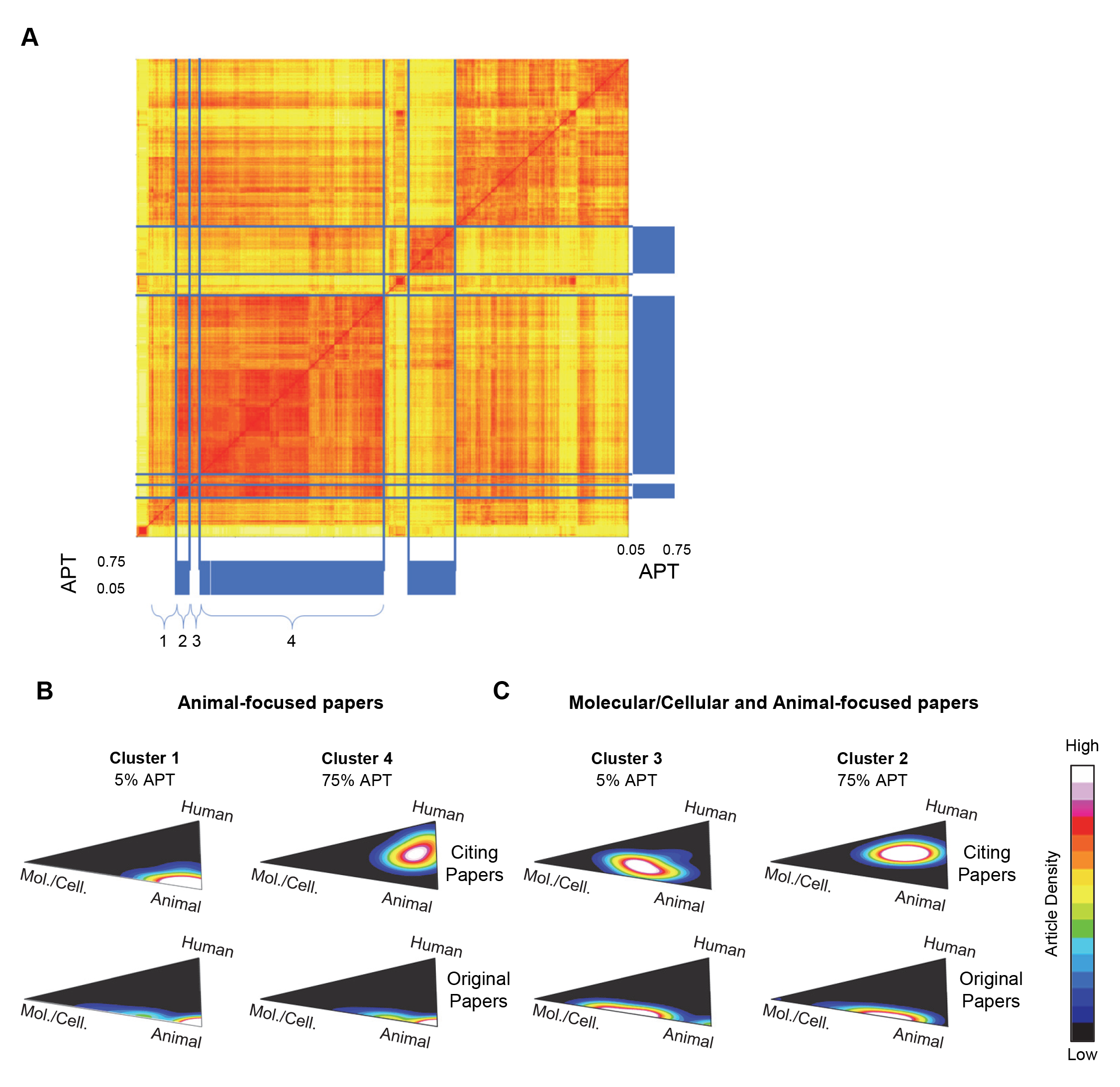

Supplement: S7 Fig — (A) Heatmap of the distance matrix for a random sample of fundamental papers from the 5% APT score and 75% APT score categories, according to the similarity of their article and citing network properties, arranged by hierarchical clustering. Papers from these 2 APT score bins appeared in the hierarchical clustering as discrete groups of consecutive, similar papers (APT score in blue on the x- and y-axes of the similarity matrix). These groups constitute common knowledge flow trajectories that have different measurable outcomes (APT score) with respect to clinical translation. Four groups are bracketed and their properties visualized. (B, C) Two sets of clusters from unsupervised hierarchical clustering are composed of similar articles (panel B, predominantly Animal-focused papers; panel C, predominantly Molecular/Cellular and Animal-focused papers) but very different APT scores. In both cases, the papers in the 75% APT score cluster are cited by papers that are more human-focused than their counterparts in the 5% APT score cluster. (TIF) [file pbio.3000416.s010.tif]

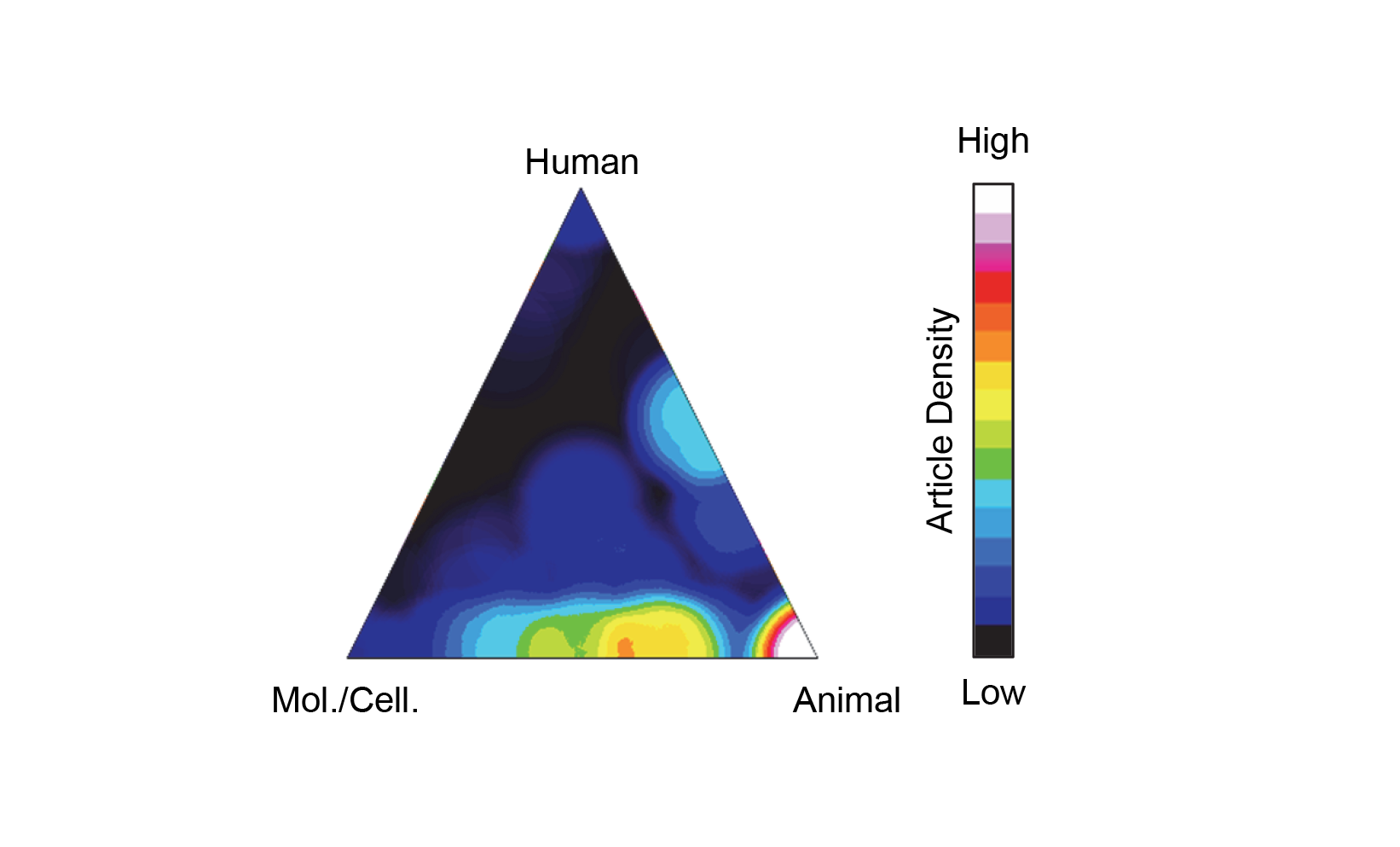

Supplement: S8 Fig — Density graph of HAMC coordinates for the 270 publications citing at least one of the 4 seminal papers leading to the 2017 Nobel Prize for Physiology or Medicine on the topic of circadian rhythms (PMIDs: 16593450, 6435882, 6094014, and 6440029). (TIF) [file pbio.3000416.s011.tif]
